# An Easy-to-Fabricate Microfluidic Shallow Trench Induced Three-Dimensional Cell Culturing and Imaging (STICI3D) Platform

**DOI:** 10.1021/acsomega.1c05118

**Published:** 2022-03-02

**Authors:** Umut Can Coskun, Funda Kus, Ateeq Ur Rehman, Berna Morova, Merve Gulle, Hatice Baser, Demet Kul, Alper Kiraz, Kemal Baysal, Ahmet Erten

**Affiliations:** † Faculty of Aeronautics and Astronautics, Istanbul Technical University, Istanbul 34469, Turkey; ‡ Department of Biomedical Sciences and Engineering, Koç University, Istanbul 34450, Turkey; § Biomedical Eng. Technology Program, Foundation University Islamabad, Islamabad Phase-I, DHA, Pakistan; ∥ Department of Physics, Koç University, Istanbul 34450, Turkey; ⊥ Department of Electronics and Communication Engineering, Istanbul Technical University, Istanbul 34469, Turkey; # School of Medicine, Department of Biochemistry, Koç University, Istanbul 34450, Turkey; ∇ Department of Electrical and Electronics Engineering, Koç University, Istanbul 34450, Turkey; ○ KUTTAM, Research Center for Translational Medicine, Koç University, Istanbul 34450, Turkey

## Abstract

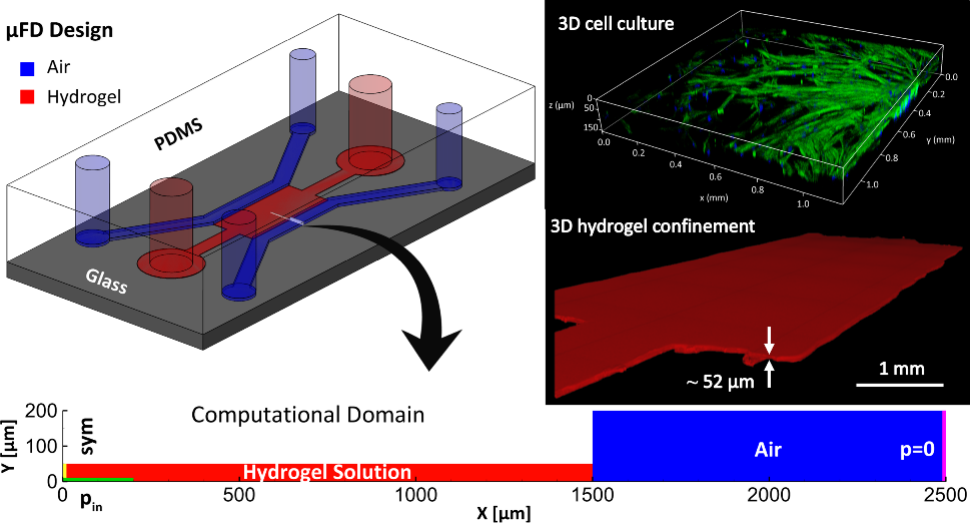

Compared to the established monolayer approach of two-dimensional cell cultures, three-dimensional (3D) cultures more closely resemble in vivo models; that is, the cells interact and form clusters mimicking their organization in native tissue. Therefore, the cellular microenvironment of these 3D cultures proves to be more clinically relevant. In this study, we present a novel easy-to-fabricate microfluidic shallow trench induced 3D cell culturing and imaging (STICI3D) platform, suitable for rapid fabrication as well as mass manufacturing. Our design consists of a shallow trench, within which various hydrogels can be formed in situ via capillary action, between and fully in contact with two side channels that allow cell seeding and media replenishment, as well as forming concentration gradients of various molecules. Compared to a micropillar-based burst valve design, which requires sophisticated microfabrication facilities, our capillary-based STICI3D can be fabricated using molds prepared with simple adhesive tapes and razors alone. The simple design supports the easy applicability of mass-production methods such as hot embossing and injection molding as well. To optimize the STICI3D design, we investigated the effect of individual design parameters such as corner radii, trench height, and surface wettability under various inlet pressures on the confinement of a hydrogel solution within the shallow trench using Computational Fluid Dynamics simulations supported with experimental validation. We identified ideal design values that improved the robustness of hydrogel confinement and reduced the effect of end-user dependent factors such as hydrogel solution loading pressure. Finally, we demonstrated cultures of human mesenchymal stem cells and human umbilical cord endothelial cells in the STICI3D to show that it supports 3D cell cultures and enables precise control of cellular microenvironment and real-time microscopic imaging. The easy-to-fabricate and highly adaptable nature of the STICI3D platform makes it suitable for researchers interested in fabricating custom polydimethylsiloxane devices as well as those who are in need of ready-to-use plastic platforms. As such, STICI3Ds can be used in imaging cell–cell interactions, angiogenesis, semiquantitative analysis of drug response in cells, and measurement of transport through cell sheet barriers.

## Introduction

In diverse fields such as basic research, drug development, and regenerative medicine, the established experimental approach to study cells is culturing them on a surface. This monolayer culture approach, in which cells are maintained on polystyrene surfaces in cell culture flasks, is termed two-dimensional (2D) culture. Although established and straightforward, these systems allow for cell–cell interactions only in 2D and do not recapitulate the complex cellular interactions found in native tissues.^1^

Growing cells in three-dimensional (3D) structures more closely resembles their existence in vivo; that is, the cells interact and form clusters mimicking their organization in native tissue. This 3D organization has been shown to support the maintenance of the correct phenotype of cells. Therefore, the cellular microenvironments of these 3D cultures are more clinically relevant.^2^ In addition, 3D systems allow the experiments to be scaled down to a microscale, thus reducing the number of cells and volume of chemicals required.^3^ These 3D approaches play a vital role in filling the knowledge gap between 2D cell culture methods and in vivo animal experiments.^4,5^ Hence, there is a growing necessity for the development of in vitro platforms mimicking the physiological in vivo systems through the formation of highly structured 3D microenvironments that are reproducible and cost-effective as well as accessible for imaging with microscopy. To this aim, microfluidic platforms have led to the development of new testing methodologies for conducting in vitro studies.

Microfluidic platforms enable precise manipulation of microenvironments and high-resolution real-time monitoring of cells and small volumes of liquids in microchannel networks in 3D.^6^ In order to control and monitor the environment of 3D microculture systems, various hydrogels that can be formed in situ within the microchannels are used to support cell adhesion and growth.^7^ These hydrogels can be natural polymers (collagen, fibrin, gelatin) as well as synthetic polymers consisting of biocompatible monomers such as poly(ethylene glycol) (PEG) and poly(lactic-*co*-glycolic acid) (PLGA).^8^ Placing cells and spheroids inside or on the surface of the hydrogels in microfluidic platforms enables one to culture and optically image them in 3D for extended periods of time. Such microfluidic platforms have enabled researchers to monitor tumor angiogenesis^9,10^ and tumor extravasation^11−13^ in greater detail and accuracy. Furthermore, 3D-functional and perfusable microvascular networks composed of human endothelial cells and bone marrow-mesenchymal stem cells (BM-hMSCs) have been formed using microfluidic platforms.^14^

Although the materials and methods used for the fabrication of microfluidic cell culture platforms vary, soft lithographical fabrication with polydimethylsiloxane (PDMS) is the most common, since it allows for rapid prototyping. PDMS-based cell culture platforms also benefit from the advantageous material properties of PDMS; that is, PDMS is optically transparent, gas permeable, and can be easily bonded to other transparent materials such as glass.^15,16^ Earlier PDMS platforms have included integrated posts, commonly referred to as “micropillars”, between channels to ensure the confinement and support the stability of hydrogels.^13,17,18^ In such a design, there exist two or more microchannels all having individual inlets and outlets that are isolated from each other with a middle channel, where a hydrogel such as collagen is confined and mechanically supported by micropillars placed in certain shapes and separations acting as burst valves. After cells are seeded and cultured on the hydrogel, various molecules and chemicals can be added to the adjacent side channels. The communication between the side channels is limited to diffusion and convection through the hydrogel formed within the middle channel acting as a porous medium. The concentration, pressure gradients, and surface tension in such a platform can be controlled to stimulate the cells in real time from the side channels.^19^ The response of cells to these stimulations such as cell proliferation, migration into the gel, angiogenesis, vasculogenesis, metastasis, and spheroid growth can be monitored using a confocal microscope with high resolution and in real-time.^20−23^ However, micropillar-based 3D cell culture platforms have two major limitations. First, the surface area where chemicals can be exchanged between the central hydrogel and side channels is limited by the need for frequent placement of micropillars.^20^ Second, parameters such as the distance between the micropillars and the design of a micropillar’s cross-section have drastic effects on the confinement of hydrogel within the middle channel and require sophisticated microfabrication facilities. This complex design may also limit the adoption of materials besides PMDS and the applicability of mass manufacturing methods such as injection molding and hot embossing.

To overcome these limitations, various approaches have been reported in recent years. Lam et al.^24^ demonstrated liquid confinement using a surface-tension-based liquid guide in a microfluidic channel by printing a hydrophilic path on hydrophobic substrates and vice versa. They reported that the confinement of liquid along the printed guides depends on the aspect ratio of a liquid cross-section and the surface wettability. They confined the liquid on the guide via a sudden change in the surface contact angle. Similar to the work of Lam et al.,^24^ Lee et al.^25^ confined the liquid along multiple guides due to the sudden change in the capillary force by means of a sudden expansion in the channel geometry. They also demonstrated the effects of height and the width of the confined liquid on the success rate of the liquid confinement along their guides. Hwang et al.^26^ proposed a rapid prototyping method for a PDMS-based microfluidic device, where confinement of liquid is achieved by capillary action by using multilayered adhesive tapes for mold production. Tung et al.^27^ also developed a microfluidic platform with a collagen hydrogel residing in a central channel. They confined the hydrogel solution using 5 × 10 μm cross-sectioned ridges, which increase the difference between advancing and receding contact angles to provide guidance. Lee et al.^28^ describe an injection-molded plastic array 3D culture platform (IMPACT), a circular-shaped well array with capillary guided hydrogel loading. The top surface of this circular platform is divided into two halves by a rail and open for easy access. A collagen solution is loaded from either half, confined around the corners and in the rail by capillary action. Although this design makes the hydrogel more accessible and is simpler than using micropillars, real-time control of chemical gradients and pressure in the cartridge is much harder. Another limitation of this system is the requirement of larger liquid volumes in the side channels (several hundred microliters) in comparison to 10–20 μL in typical polydimethylsiloxane microfluidic platforms.^18,29^

In our study, we present a novel, easy-to-fabricate, microfluidic shallow trench induced three-dimensional cell culturing and imaging (STICI3D) platform, suitable for both rapid-fabrication and mass-manufacturing methods. STICI3D has a simple geometry for 3D cell cultures inside or on the surface of a hydrogel confined within a shallow trench fully in contact with two side channels for precise real-time control of a cell microenvironment such as chemical gradients and pressure. In our design, by achieving hydrogel confinement without necessitating micropillar-based burst valves, we significantly simplified the device design and maximized the surface area through which the hydrogel exchanges media with the side channels. The capillary-based STICI3D design allows it to be fabricated with soft lithography using molds prepared with adhesive tapes and razors alone, which supports the easy adoption of materials and methods for mass production such as hot embossing and injection molding as well. Moreover, STICI3D requires only small volumes of hydrogel (∼3 μL) or chemicals.

To streamline the STICI3D design and development, a meticulous investigation of the effect of design parameters on the confinement of hydrogel solution within the shallow trench was achieved with Computational Fluid Dynamics (CFD) simulations, which allow for a numerical evaluation of microfluidic cell culture system designs along with their respective flow rates and patterns prior to fabrication.^30^ In this study, we employed a numerical setup compatible with similar flow problems reported in the literature and our previous studies.^31,32^ We investigated the effects of individual design parameters such as corner radii, channel height, and selection of surfaces with different wettability under various loading pressures on the confinement of a hydrogel solution within the shallow trench numerically using CFD simulations supported with experimental validation. To assay the compatibility of STICI3D with cell culture, we examined the attachment and proliferation of human mesenchymal stem cells in the central collagen channel. Finally, we also demonstrated that human umbilical cord endothelial cells (HUVEC) lining the collagen respond to the molecules diffusing through the hydrogel.

This article is organized as follows. In the following section, after the STICI3D design is introduced, the CFD-based numerical model and experimental setup is explained. In the order of presence; verification and validation of the numerical method and results of numerically and experimentally investigated design parameters on the confinement of liquid within the shallow trench are presented in the subsections, followed by the experimental results of exemplary 3D cell cultures. Finally, conclusions are discussed in the final section.

## Materials and Methods

### Design of STICI3D Platform

**Figure 1:**
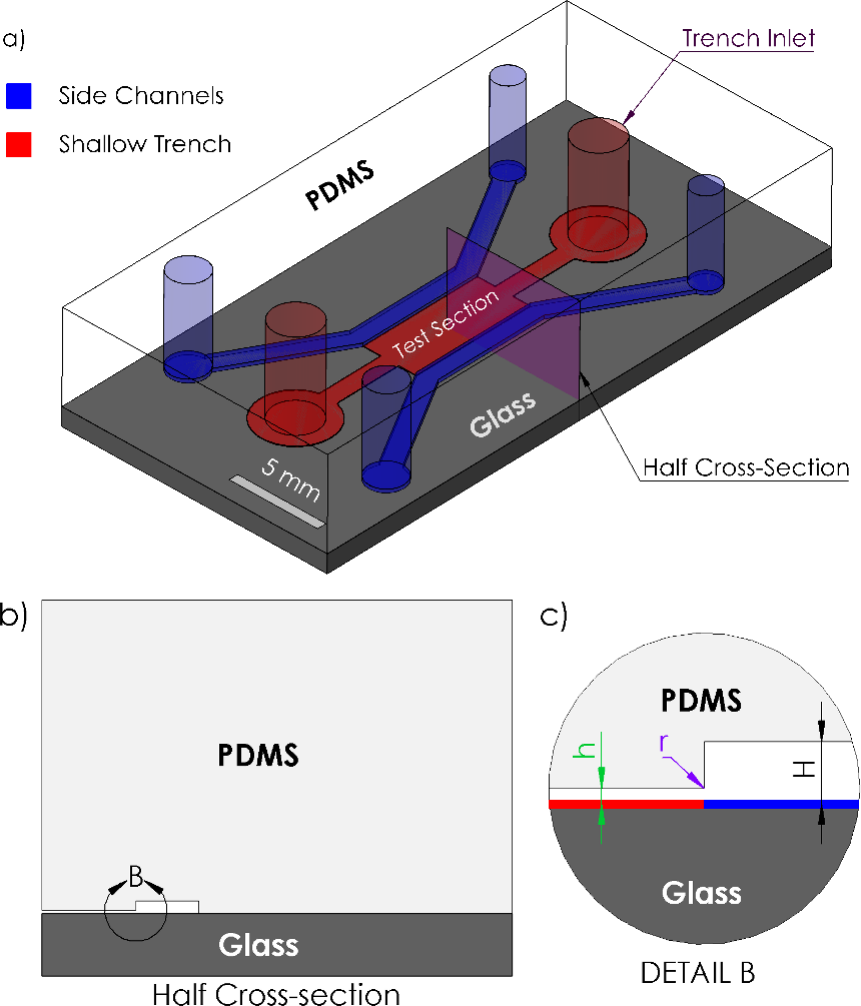
(a) 3D geometry of STICI3D, (b) half cross-section view of the STICI3D at the midsection, and (c) detailed view of the cross-section at trench to the side channel joint.

The proposed simple design of the STICI3D platform consists of two side channels (shown in blue in Figure 1a) and one shallow trench in the middle (shown in red in Figure 1a), each having individual inlets and outlets. The inlet and outlet chambers for the shallow trench were designed to be 4 mm in diameter and the same height (*h*) as the trench in order to reduce the confinement problems during the hydrogel loading. To further reduce the end-user dependent variations in loading the hydrogel solution, the diameter of the inlet for the shallow trench was designed to be larger than the diameter of the pipet tip to keep the liquid loading pressure close to atmospheric pressure. The inlet diameter of the trench in STICI3D was reduced when a higher inlet pressure was required for experimental testings of the parameters (The 2D sketch of the design can be seen in Figure S1).

The geometry of the STICI3D platform is one continuous chamber with the height “*h*” of the shallow trench (shown in red in Figure 1a) being shorter than the height “*H*” of the adjacent side sections shown in blue. Figure 1b shows the half cross-section of the STICI3D microfluidic platform at midplane highlighted in Figure 1a. The detailed view of this cross-section at the vicinity of trench to the side channel joint, marked as detail B, is shown in Figure 1c. The geometry of the test section of the STICI3D is constrained to have a constant cross-section as shown in Figure 1c.

In order to identify the range of STICI3D design parameters for a successful confinement of the liquid within the trench, design parameters such as the height of the trench *h*, selection of hydrophilic surfaces, the contact angle of the hydrophilic surfaces, and radii *r* at the corners of the trench were investigated both numerically and experimentally where possible. An experimental investigation of some design parameters was impractical due to difficulties such as obtaining the desired surface contact angles. Therefore, for those impractical cases the prediction of liquid confinement was limited to CFD simulations only. To identify safer design values against the overflow of liquid into side channels, which might result from possible manufacturing or end-user dependent failures, STICI3D designs that successfully confined the liquid at 0 Pa loading pressure were also investigated numerically using higher loading pressures, and these designs were validated experimentally where possible.

### Numerical Setup

A finite volume-based commercial software Ansys Fluent was used as the flow solver to investigate the two-phase flow of immiscible fluids. The simulations were designed as two-phase, transient, incompressible, 2D, and laminar. The volume of fluid method^33^ (VOF) was used to track the interface between two immiscible fluids, namely, air and liquid, where the Continuum Surface Stress method with wall adhesion was chosen to model the surface tension and adhesion.

Collagen Type I was selected as the hydrogel to be tested in our STICI3D design studies. The contact angles of deionized water and hydrogel solution on the surface of PDMS, plain glass, and poly-d-lysine (PDL)-coated glass (PDLcG) in a medium of air were experimentally obtained at the early phases of the study (see Section 1.4 in the Supporting Information). Since the difference in contact angles of water and hydrogel solution were less than 10° for all of these surfaces, water and hydrogel solution were assumed to have similar wetting behavior for the tested material surfaces. Considering that deionized water is cheaper than collagen, most of the experiments in the STICI3D design study and, therefore, numerical studies were conducted with deionized water as the liquid phase. In all simulations, the liquid is modeled as Newtonian, where its density and viscosity were selected constant and the same as those of water.

Even though the problem had a steady nature, since there was no distinct and steady inflow, the simulations were conducted as transient. An explicit VOF formulation with a sharp interface modeling was chosen for high accuracy. Second-order upwind and geo-construct spatial discretization schemes were again preferred to have high accuracy for the momentum and volume fraction, respectively. The downside of the explicit formulation was that it had a Courant-based time step size constraint. A dynamic adaptive mesh refinement and variable time-stepping methods were employed to reduce the required number of cells to a minimum and to keep the maximum Courant Number lower than 0.25, which yields a varying time step size in the order of 10^–8^ s as a result of the adapted mesh sizes in most of the cases of this study.

#### Computational Mesh, Boundary Conditions, and Solver Settings

**Figure 2:**
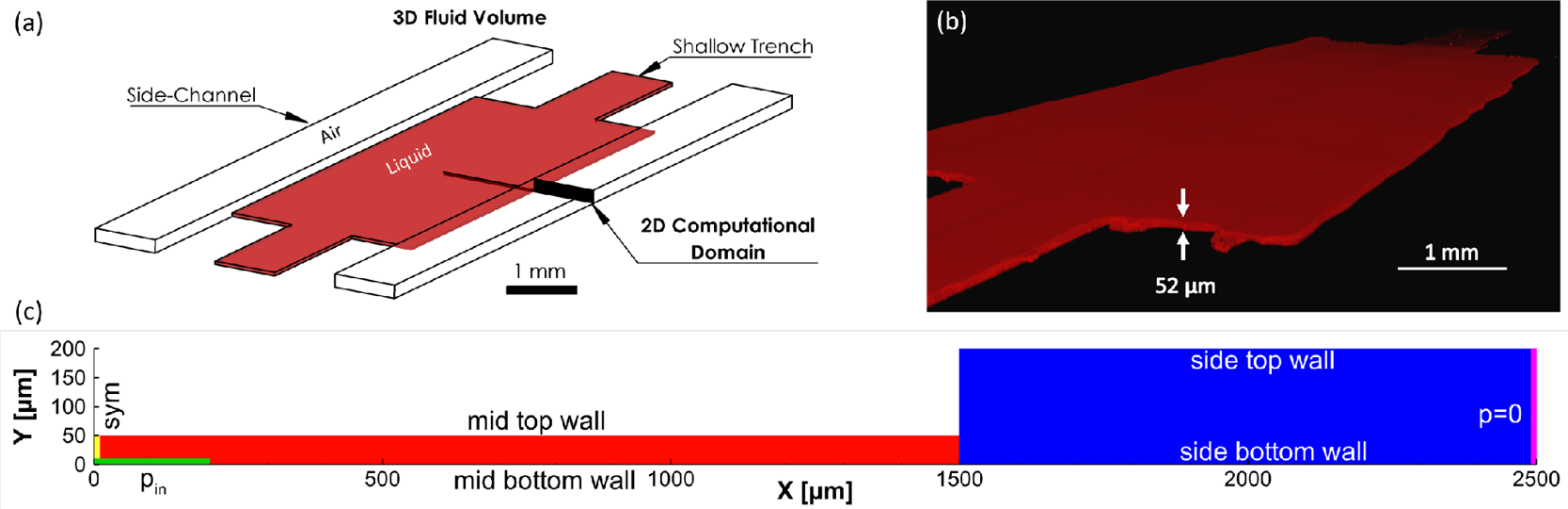
(a) 3D volume of fluid inside the STICI3D and 2D computational domain chosen to investigate confinement of liquid within the trench shown in red, (b) 3D profile of hydrogel colored with Rhodamine B, imaged with a confocal microscope using a z-stack function, (c) 2D computational domain, initial and boundary conditions. Red and blue areas represent liquid and air phases, respectively. Faces with symmetry, pressure inlet, and pressure outlet boundary types are highlighted in yellow, green, and pink, respectively. All boundaries that are not highlighted are treated as a wall with no-slip boundary condition.

A full 3D representation of the problem in CFD calculations was first attempted; however, this approach was found to be impractical due to high computational costs. Extremely fine mesh sizes were required in the vicinity of the air–liquid interface, which also forces the time step size to be very small. The resulting 3D computational mesh was predicted to have 100 million cells and was expected to run for at least 10^5^ time steps for each case. However, the length of the trench is 2 orders of magnitude larger than its height (see Figure S1 in the Supporting Information), where the liquid is assumed to take the shape of this area and be confined, suggesting that a 2D representation is valid for a variety of parametric numerical investigations. Therefore, instead of modeling the flow inside the whole STICI3D in 3D, a cross-section of the fluid volume in the middle of the channel highlighted in purple in Figure 1a was selected as the computational domain. Given the symmetrical nature of the problem, half of the cross section was taken as the computational domain to further reduce the computational costs. Both the 3D fluid volume and the chosen 2D computational domain are shown in Figure 2a.

In this two-phase flow problem, air is selected as the carrying fluid, and water is selected as secondary. Since the main concern of the simulation is to predict the final shape of the interface and the condition of the confinement of the liquid within the shallow trench, the volume fraction of water within the trench is initialized as 1 in all cases, which indicates the trench is initially filled with liquid. The inlet and outlet sections of the actual 3D channel are out of the selected 2D computational domain. However, in order to model this flow problem in 2D, the required inlet and outlet boundaries were placed sufficiently away from the air–water interface. The details of the 2D computational domain, as well as the initial and boundary conditions of the flow problem, are shown in Figure 2c. The inlet of water and outlet of air were highlighted in green and pink, respectively. The left vertical boundary highlighted in yellow was selected as a symmetry boundary type. The top and bottom boundaries were treated as walls with a no-slip boundary condition.

**Figure 3:**
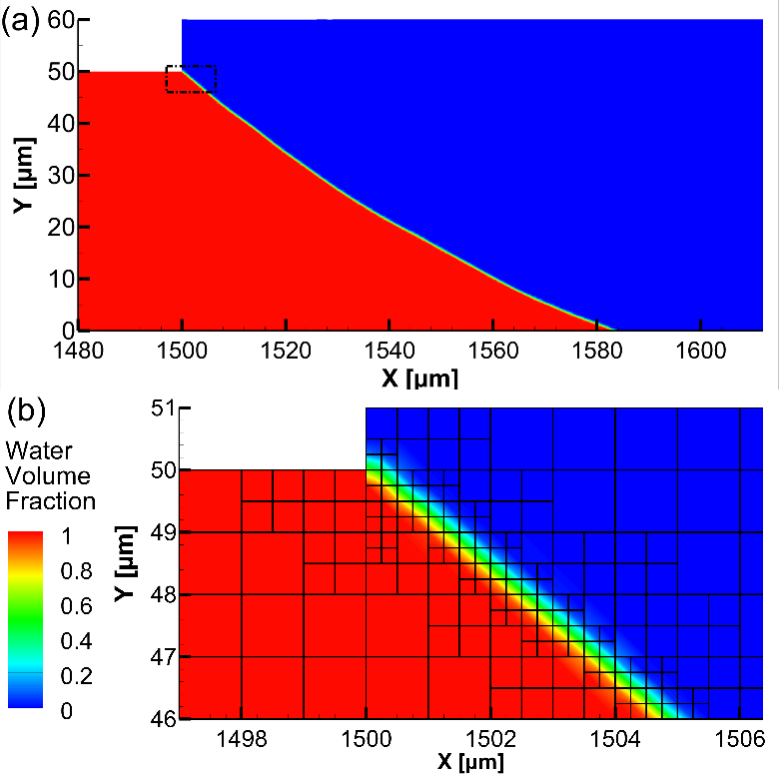
(a) Water volume fraction contours near the air–water interface at the end of the simulation and (b) adaptive mesh around the air–liquid interface in the rectangular area indicated by the dashed line in (a).

The 2D geometry was initially meshed with uniform square cells. A dynamic adaptive mesh refinement in the vicinity of the air–liquid interface was applied to accurately predict the shape of the interface and the condition of the confinement. The final shape of the interface and a representative mesh in the vicinity of the interface is shown in Figure 3.

A reference case representing our typical design parameters was selected for the verification and validation of our numerical work. In this reference case the heights of the trench and side channels were chosen as *h* = 50 μm and *H* = 200 μm, respectively, where the material of the whole bottom wall was selected as PDLcG. Experimentally obtained hydrogel solution contact angles of 31° and 107°, as reported in the Supporting Information, Section 1.4, were employed, respectively, on PDLcG and PDMS walls. The details of the whole verification and validation study are explained in detail in the following subsection (see section: Verification and Experimental Validation of Numerical Results).

### Experimental Setup

#### Mold and Device Fabrication

In order to experimentally validate the CFD model and further support the design with experiments, STICI3D devices were fabricated out of PDMS elastomer using soft lithography, and they were plasma-bonded to PDL-coated glass microscope slides. The PDL coating on the glass provided lower contact angles. The molds for soft lithographic replication were prepared by utilizing layers of adhesive polyimide (Kapton) and aluminum tapes to provide height differences between the side channels and the shallow trench (see the Supporting Information, Section 1.2). To obtain a 50 μm trench height (*h*) and 200 μm side channel heights (*H*), a layer of aluminum tape with an inherent thickness of 150 μm was laid over the first patterned layer of polyimide tape with a thickness of 50 μm.

The first layer of adhesive tape (polyimide) was laid over a sheet of acetate paper and cut into the desired pattern followed by stacking up the next layer of adhesive tape (aluminum) on top of the previously cut adhesive tape and cutting into the new desired pattern. Design patterns (see the Supporting Information, Section 1.1) were cut either manually by a razor or by a digital craft cutter (Silhouette Cameo, Silhouette America Inc.). Figure 5a displays a picture of the produced mold. Keeping the side channel height as *H* = *h* + 150 μm, STICI3Ds with trench heights (*h*) of 100, 150, and 300 μm were produced with the same method (see the Supporting Information, Section 1.2).

PDMS elastomer (Sylgard 184, the Dow Chemical Company) base and curing agent were mixed by a weight ratio of 10:1. The mixture was thoroughly stirred and desiccated until all the air bubbles were removed. The mixture was poured into the molds, and the molds were left at a constant temperature of 55 °C for 3 h for curing. After the cured PDMS was demolded, the side channel inlet and outlets were formed using biopsy punchers with 1.5 mm and 2 mm core size diameters, respectively. Both trench inlet and outlet were punched with a puncher of diameter 2.5 mm. For the cases where high loading pressures were tested, the inlet diameter of the trench was lowered to 1.5 mm. Heights of the STICI3D trench and side channels on the demolded PDMS part were measured by a stylus profilometer (Dektak, Bruker Corp.) (see the Supporting Information, Section 1.3). PDMS and precleaned PDL-coated glass parts (PDLcG; Menzel Gläser Polysine slides, Thermo Scientific) were bonded together immediately after the surface activation by oxygen plasma for 60 s with 100 W radiofrequency (RF) power under vacuum. After the bonding, postcuring was applied by placing the STICI3Ds in an oven at 65 °C for 1 h to recover hydrophobicity and to enhance the bonding strength. STICI3Ds used in the experiments were all processed by postcuring, unless otherwise stated.

#### Preparation and Formation of Hydrogel

The collagen hydrogel recipe was reformulated based on a recipe from previously published protocols.^34,35^ The ingredients used for the collagen hydrogel preparation are listed in the order of pipetting in Table 1. All ingredients were stored at 4 °C before usage and kept on ice during mixing. 10X Dulbecco’s Modified Eagle’s Medium (DMEM, F-12 Ham with 15 mM HEPES and sodium bicarbonate, without l-glutamine, Sigma-Aldrich Inc.), NaOH (1 M, sodium hydroxide, Sigma-Aldrich), NaHCO_3_ (7.5% w/v, sodium hydrogen carbonate, Sigma-Aldrich), and 1X DMEM solutions and collagen (Type-I, bovine, 6 mg/mL, Sigma-Aldrich Inc.) were mixed gently to initiate gel formation. Immediately after mixing, a certain volume of the mixture depending on the trench height was pipetted from the inlet of the trench. After the collagen-based hydrogel solution was confined within the trench of the STICI3D, the devices were left for gelation for ∼1 h in an incubator held at 37 °C. After hydrogel formation, the STICI3D was ready for cell seeding. For imaging purposes, the hydrogel was dyed with a fluorescent dye. Rhodamine B was dissolved in deionized water with a concentration of 1 mM and was kept at 4 °C before preparing the hydrogel solution. Four microliters of Rhodamine B (9-(2-carboxyphenyl)-6-(diethylamino)-*N*,*N*-diethyl-3*H*-xanthen-3-iminium chloride) solution was mixed with 26 μL of 1X DMEM prior to mixing, constituting ∼5% of the final volume of the hydrogel solution.

**Table 1:** Collagen Hydrogel Ingredients

reagents	volume
10X DMEM	5 μL
NaOH 1M	0.5 μL
NaHCO_3_ (7.5% w/v)	5 μL
1X DMEM	30 μL
collagen Type I (6 mg/mL)	40 μL

#### Confinement Tests

Because of the similar wettability of collagen solution and water, confinement of the liquid in the shallow trench of STICI3D was iniatially observed by administering a solution of Rhodamine B dye dissolved in water. A plasma treatment renders the PDMS surface hydrophilic. Therefore, the confinement of water dyed with Rhodamine B was first tested immediately after the PDMS and PDLcG parts were bonded upon plasma treatment when the surfaces were still hydrophilic. A 50 μm trench height (*h*), as in the reference case, was selected for the confinement test of the STICI3D with hydrophilic walls (see the Supporting Information, Section 1.5 and Movie S1). Second, to promote the hydrophobic recovery of the surfaces, STICI3Ds were left in the oven at 65 °C for 1 h after assembly as mentioned in a previous section (see the section: Mold and Device Fabrication). Water confinement tests after the hydrophobic recovery were conducted with 0 Pa inlet pressure cases for STICI3Ds with trench heights of *h* = 50, 100, 150, and 300 μm.

In order to observe the confinement under inlet pressures higher than 0 Pa during a liquid loading, a STICI3D with a trench height *h* of 50 μm was produced with a narrower inlet to the trench so that the conical tip of the micropipette sealed the inlet entirely (see the section Mold and Device Fabrication).

Following the successful confinement tests with water, hydrogel solution confinement within the resulting STICI3D platform was investigated. Rhodamine B fluorescent dye was introduced into the hydrogel mixture as described in a previous section (see the section Preparation and Formation of Hydrogel). A STICI3D with a trench height *h* of 50 μm and side channel height *H* of 200 μm was prepared for hydrogel solution loading. The hydrogel confinement in the shallow trench was observed under an inverted fluorescence microscope (Zeiss Axio Observer Z1) using a 10× objective. Results of these tests are presented in detail in the following sections (see the section Collagen Confinement Tests on Resulting STICI3D).

#### Cell Studies—Human Mesenchymal Stem Cell Growth on the Collagen Hydrogel

Various types of cells can be seeded into the hydrogel. Among these, mesenchymal stem cells (MSCs) are a subgroup of stem cells that reside in various tissues and are capable of differentiation into different cell types.^36^ Recent studies have demonstrated the utility of MSCs as an alternative human cell source that can be used in engineered platforms recapitulating different human tissues and organs.^37^ A 3D culture of MSCs affects various properties of these cells, such as phenotype,^38^ the proteins that are secreted from these cells,^39^ and their differentiation.^40^ Hence, MSC chemotaxis has been quantitatively studied in microfluidic devices.^41^

To demonstrate that the designed STICI3D is suitable for a 3D culture and monitoring of cells, human mesenchymal stem cells (hMSCs) isolated from umbilical cords were utilized (see Section 1.6 in the Supporting Information). According to previously established methods, the cords were used to form explant cultures of hMSCs.^42^ Cell outgrowths from explants were maintained in DMEM: Nutrient Mixture F-12 containing 10% fetal bovine serum (FBS) (Biowest), 1% l-glutamine (Gibco), and 0.1% penicillin/streptomycin (Gibco) (culture medium). The culture dishes were maintained in a 5% CO_2_ incubator at 37 °C. To enable the cells to feed and proliferate efficiently, the cell medium was changed every 3 d.

Adherent hMSCs were detached from the plates by incubating the samples with a 0.05% trypsin-EDTA (EDTA = ethylenediaminetetraacetic acid) solution (Gibco) for 5 min at 37 °C. The cells were suspended in the culture medium and counted using a hemocytometer, and 20.000 hMSCs in 10 μL of culture medium were added to one of the side channels of an STICI3D, in which a collagen hydrogel was formed within the shallow trench as described above. Following this, the STICI3D was kept upright for 4 h in a 5% CO_2_ incubator at 37 °C to facilitate the cell attachment onto the collagen hydrogel. Thereafter, both side channels were filled with culture medium, and this was refreshed every other day.

hMSCs placed on collagen hydrogels were fixed after 3–6 d in the culture. Cells were fixed with 4% paraformaldehyde in phosphate-buffered saline (PBS) and subsequently stained with phalloidin-FITC (fluorescein isothiocyanate) (1:200) and with 2-(4-amidinophenyl)-1*H*-indole-6-carboxamidine (DAPI) antifade solution (Abcam) (1:200). The stained cells within the STICI3D were imaged using an inverted confocal microscope (Leica DMi8 SP8) with a 10× objective.

#### Cell Studies—Culture of Human Umbilical Cord Endothelial Cells on the Collagen Hydrogel and Treatment with TNF-α

The collagen was loaded onto the STICI3Ds as previously described (final collagen concentration: 2.98 mg/mL). After 1 h at 37 °C incubator, HUVEC cells (ATCC) grown in culture medium (culture medium: ECGM (Lonza) + 5% FBS, penicillin/streptomycin (1000X solution, Gibco) + endothelial cell growth supplement; ECGS, Sigma) were trypsinized (Trypsin-EDTA, Thermo), counted, and seeded (20 000 cells per middle channel) on the collagen hydrogel. The STICI3Ds were placed vertically for 4 h to promote cell attachment onto the hydrogel surface. The culture medium was introduced to both side channels and was further incubated for 24 h. Tumor necrosis factor alpha (TNF-α, PeproTech) was introduced at the side channel opposite to the channel containing endothelial cells at 1 μg/mL concentrations in culture medium. After 24 h of incubation in the incubator, the live cells were imaged by loading cells with the live-cell imaging dye; calcein–a live cell stain. It is introduced to the cells in the acetoxymethyl ester form (Calcein-AM, Invitrogen); once the ester form enters the cell, these groups are cleaved, and the free form is fluorescent in live cells. Upon addition to both side channels, the dye was incubated for 30 min, enabling access to the cytoplasm. The nonloaded dye was washed with the culture medium. Images of live cells were taken by a Leica confocal microscope (Leica DMi8 SP8) in the live cell imaging mode under 5% CO2 and at 37 °C.

## Results and Discussion

### Mold and Device Fabrication

The multilayer adhesive tape-based mold fabrication presented allowed a reliable, rapid, and easy method for the fabrication of multilevel channel structures. The utilization of a sheet of acetate paper as a substrate allowed the elimination of the silanization step resulting in a significant reduction in the mold production duration. Moreover, the stacking up of the adhesive tape layers on top of each other eliminated the manual alignment requirement of different layers. The production of mold cut into two layers of adhesive tape (aluminum tape stacked on top of polyimide tape) for our STICI3D device took less than 4 min using a craft cutter. The molds were usable up to five times depending on the level of damage caused during the demolding process.

### Verification and Experimental Validation of Numerical Results

An extensive numerical verification and validation study on the reference case that was described in a previous section (see the section Computational Mesh, Boundary Conditions, and Solver Settings) was conducted. In verification study, the mesh and time step sizes were gradually reduced until the numerical results became independent of them. After the mesh and time step sizes were determined, the numerical results of the reference case were compared with the experimentally obtained data to reveal its accuracy, in the validation study.

**Figure 4:**
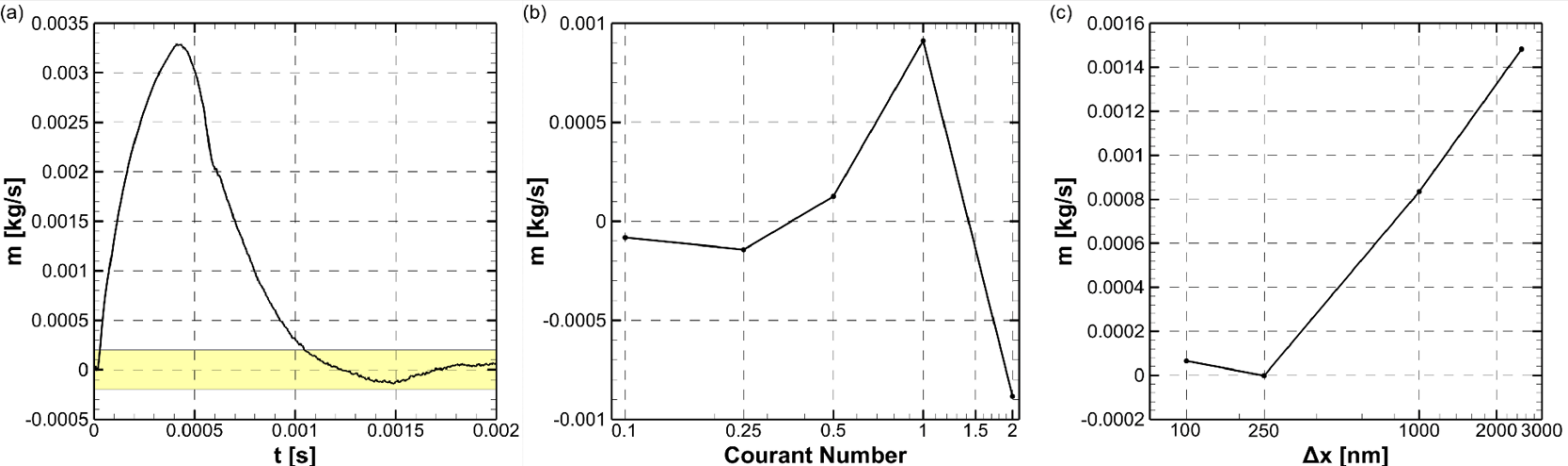
(a) Water mass flow rate from the inlet of the reference case where *h* = 50 μm, *H* = 200 μm, *r* = 0 μm, and *P*_in_ = 0 Pa, with PDLcG on middle and side bottom wall. The vicinity of zero mass flow rate indicating liquid confinement is highlighted in yellow. Verification study: (b) time step size depedency test results at 250 nm cell sizes near interface and (c) mesh dependency test results at maximum Courant Number of 0.25.

The mass flow rate from the inlet section of the computational domain is selected as the dependent flow variable for the mesh and time step size dependency tests. The change of the inlet mass flow rate for the reference case is shown in Figure 4a. The positive inlet mass flow rate indicates that liquid is sucked from the inlet section into the shallow trench. As can be seen from Figure 4a, liquid is sucked from the inlet for a duration of ∼1 ms, and the mass flow rate converged to a steady-state value of zero, meaning that liquid is confined. The mesh and time step size dependency tests were conducted by comparing the average inlet mass flow rate between 0.0015 and 0.002 s for all test cases. The cell size of the adaptive mesh at the interface and maximum allowed Courant Number were selected as independent variables. The results of the verification study are shown in Figure 4b,c. A list of maximum Courant Numbers between 0.1 and 2 and cell sizes between 125 and 2500 nm were selected for these tests. It can be seen that the solution became independent of Courant Number and mesh size, at Courant Number of 0.25 and cell size around the interface of 250 nm, respectively.

**Figure 5:**
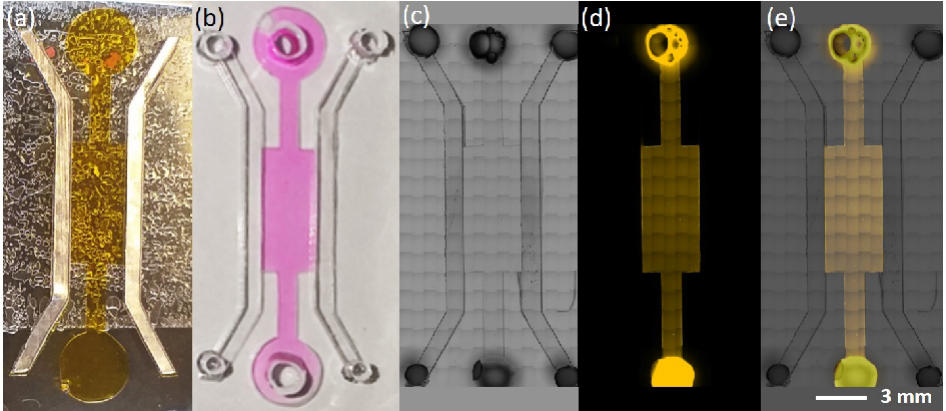
(a) Mold produced by adhesive tapes (see the Supporting Information Section 1.2). (b) Water confinement inside the produced STICI3D with trench height *h* = 50 μm and side channels *H* = 200 μm. Areas filled with Rhodamine B solution in deionized water are seen as pink (see Movie S2 in the Supporting Information). Brightfield (c), fluorescent (d), and merged (e) images of collagen confined within the trench of the STICI3D with *h* = 50 μm and *H* = 200 μm. Yellow areas show the hydrogel dyed with Rhodamine B.

Because of the small scales of the channels, the profile of the air–water interface could not be measured experimentally. The experimental validation of the study could be made by comparing the conditions of the confinement of water within the shallow trench. As mentioned in a previous section (see the section Confinement Tests), the liquid confinement experiment was performed in a STICI3D with *h* = 50 μm, using dyed water as the liquid. The result of a successful water confinement within the shallow trench is shown in Figure 5b. (See Movie S2 in the Supporting Information for continuous loading.) The numerical results shown in Figure 4a where the inlet mass flow rate converges to zero, which indicates the interface also converges to a steady-state shape shown in Figure 3a and the water is confined within the shallow trench, are in good agreement with the experimentally obtained confinement condition.

### Effect of Corner Radius

**Figure 6:**
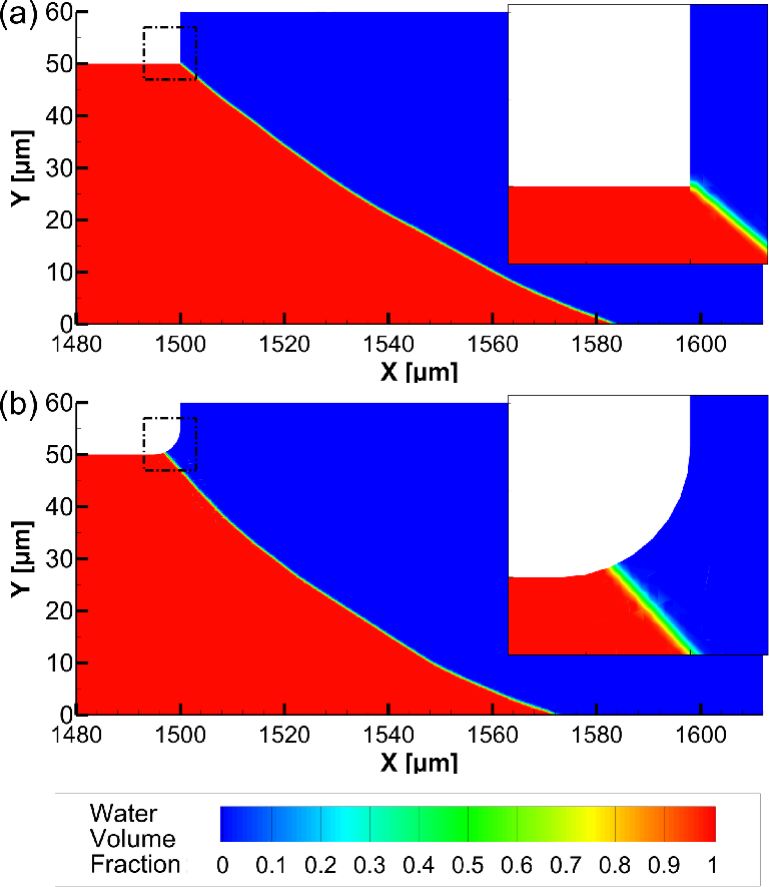
Effect of corner radius *r* on liquid confinement, water volume fraction contours of cases with (a) sharp (*r* = 0 μm) and (b) rounded corners (*r* = 5 μm), where red color corresponds to liquid phase and blue color to gas phase. A close-up image of the corner area within the dashed line is shown in the upper-right corner of each figure. *h* = 50 μm, *H* = 200 μm, *P*_in_ = 0 Pa, PDLcG on middle and side bottom walls.

Because of its small scales, it is challenging to manufacture microfluidic devices with exact sharp corners. For instance, in the current study, the corner radius *r* of a manufactured STICI3D was measured to be *r* ≈ 5 μm. In order to accommodate for limitations from a manufacturing standpoint as well, the effect of the corner radius *r* (highlighted in purple in Figure 1c) of the shallow trench, where it expands to side channels, was studied. Water volume fraction contours in the vicinity of the interface for cases with a sharp and rounded corner are shown in Figure 6a,b, respectively.

As it can be seen from Figure 6, a radius of *r* = 5 μm causes only a slight change in the interface shape and does not affect the confinement. However, since the manufactured actual geometry had a corner radius of 5 μm, the parametric investigations from this point forward were conducted with *r* = 5 μm. The effect of the corner treatment on the inlet mass flow rate is shown in the Supporting Information, Section 2.1.

### Effect of Trench Height

**Figure 7:**
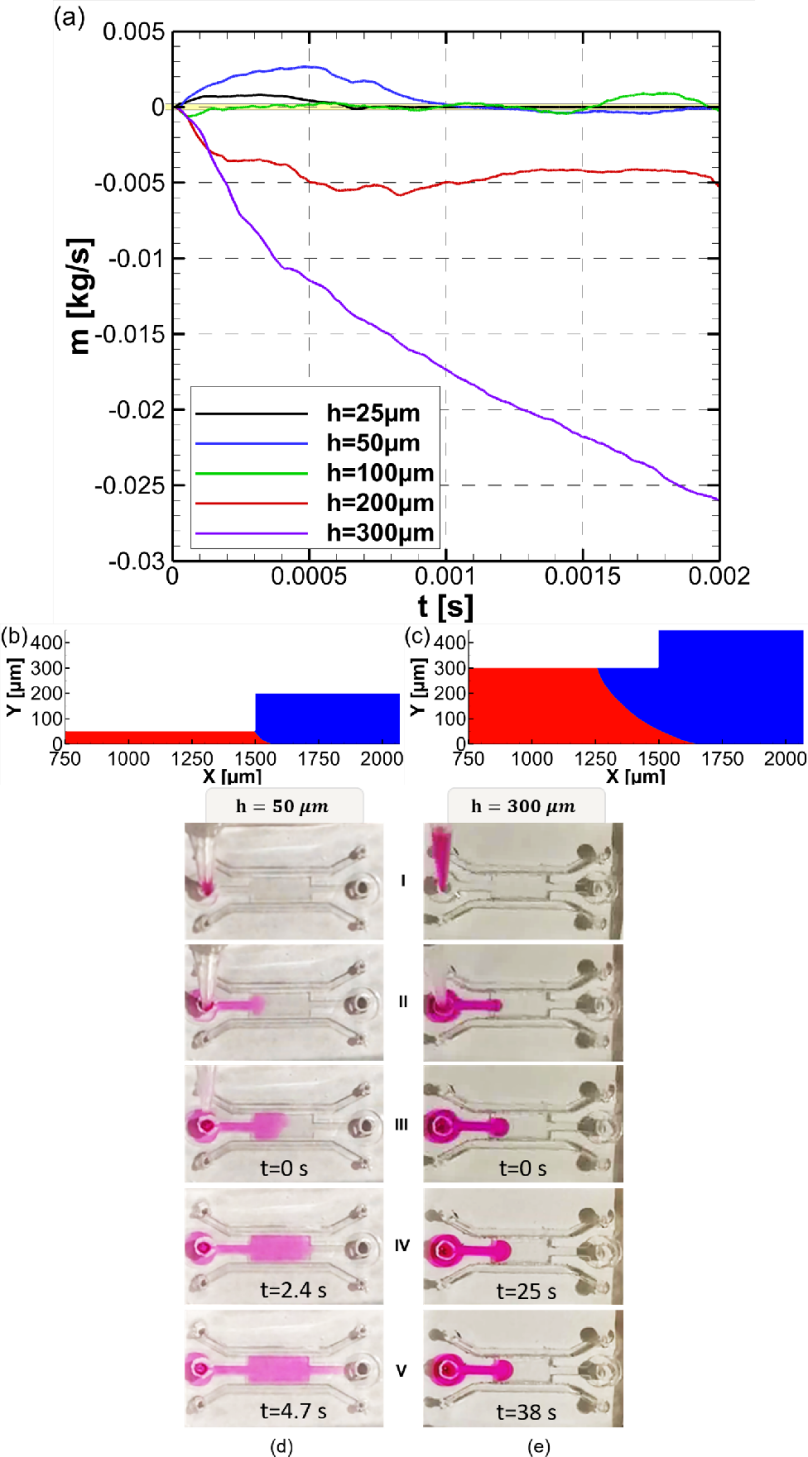
(a) Effect of trench height on water mass flow rate at the inlet boundary. *H* = *h* + 150 μm, *r* = 5 μm, *P*_in_ = 0 Pa, PDLcG on trench and side bottom walls. The vicinity of zero mass flow rate indicating water confinement is highlighted in yellow. The negative mass flow rates of *h* = 200 and *h* = 300 μm cases shows that the liquid will not be drawn into the trench by capillary action. Resulting interface shapes at *t* = 0.002 s for (b) *h* = 50 μm and (c) *h* = 300 μm. Snapshot images of loading with Rhodamine B solution in deionized water obtained for STICI3D with (d) *h* = 50 μm and (e) *h* = 300 μm. The moment when the pipet tip was retracted from the inlet of the trench is assigned as *t* = 0 s. See Movie S2 and Movie S5 in the Supporting Information for continuous recordings of the loading.

The liquid confinement within the shallow trench is maintained by the capillary forces, which are more dominant than the viscous and inertial forces in microchannels. As a consequence, the height of the trench *h* (highlighted in green in Figure 1c) becomes a critical design parameter. Therefore, trench heights from *h* = 25 μm to *h* = 300 μm were investigated numerically. The effects of trench height on the inlet mass flow rate is shown in Figure 7a.

It is observed from the simulations that, for *h* = 25 μm to *h* = 100 μm, the water is sucked into the trench and is confined. For *h* = 200 μm and above, the liquid that initially resides within the trench is pushed back to the inlet of the trench. This result indicates that the liquid will not be drawn into an initially empty trench by capillary action. Hence, in order to confine the water within the trench for more than *h* = 200 μm, the water must be pushed with an inlet pressure greater than zero. In a 3D application, although high inlet pressures can push the interface from inlet to outlet, it would also push the interface to side channels and could cause the liquid to overflow. Therefore, the height of the trench was selected as *h* = 50 μm, since it is within the range of confined cases in CFD simulations and is easier to fabricate. The interface shapes for trench heights *h* of 50 μm and *h* of 300 μm at *t* = 0.002 s are shown in Figure 7b,c (see also the Supporting Information, Section 2.2).

The numerical results were obtained from a model that has been reduced to 2D, and the inlet/outlet sections of the fluids were not in their actual positions in 3D. In order to further increase the reliability of the numerical model, an experimental investigation on the effect of the trench height was performed with STICI3Ds with trench heights of *h* = 50, 100, 150, and 300 μm. The real-time videos of liquid loading processes for STICI3Ds with *h* = 50, 100, and 150 μm trench heights are provided in Movies S2–S4 in the Supporting Information. In parallel with the numerical results, as the trench height increased, the liquid suction into and through the trench became harder and slower. Furthermore, for the trench height of *h* = 300 μm, the immediate liquid suction without an applied pressure from the inlet was not observed as it is predicted by the numerical simulations. Figure 7e demonstrates snapshot images of a liquid loading within the STICI3D with *h* = 300 μm. Also see Movie S5 in the Supporting Information for a continuous recording.

### Effect of Bottom Wall Surface Wettability

For a STICI3D with a hydrophilic bottom wall, adhesion forces pull the air–water interface from the inlet section to every lateral direction until water wets the bottom wall completely. However, the cohesion forces pull the liquid back to the inlet of the trench, and the imbalance between these forces moves the interface. Where the shallow trench ends and expands to side channels, a sudden change in the capillary effect occurs. The top edge of the interface stops at this sudden expansion region on the top wall, and the bottom edge of the interface continues to move to the side channels. The interface moves and stretches until the forces acting on the interface reach an equilibrium and confine the liquid, as it can also be seen in Figure 6a.

**Figure 8:**
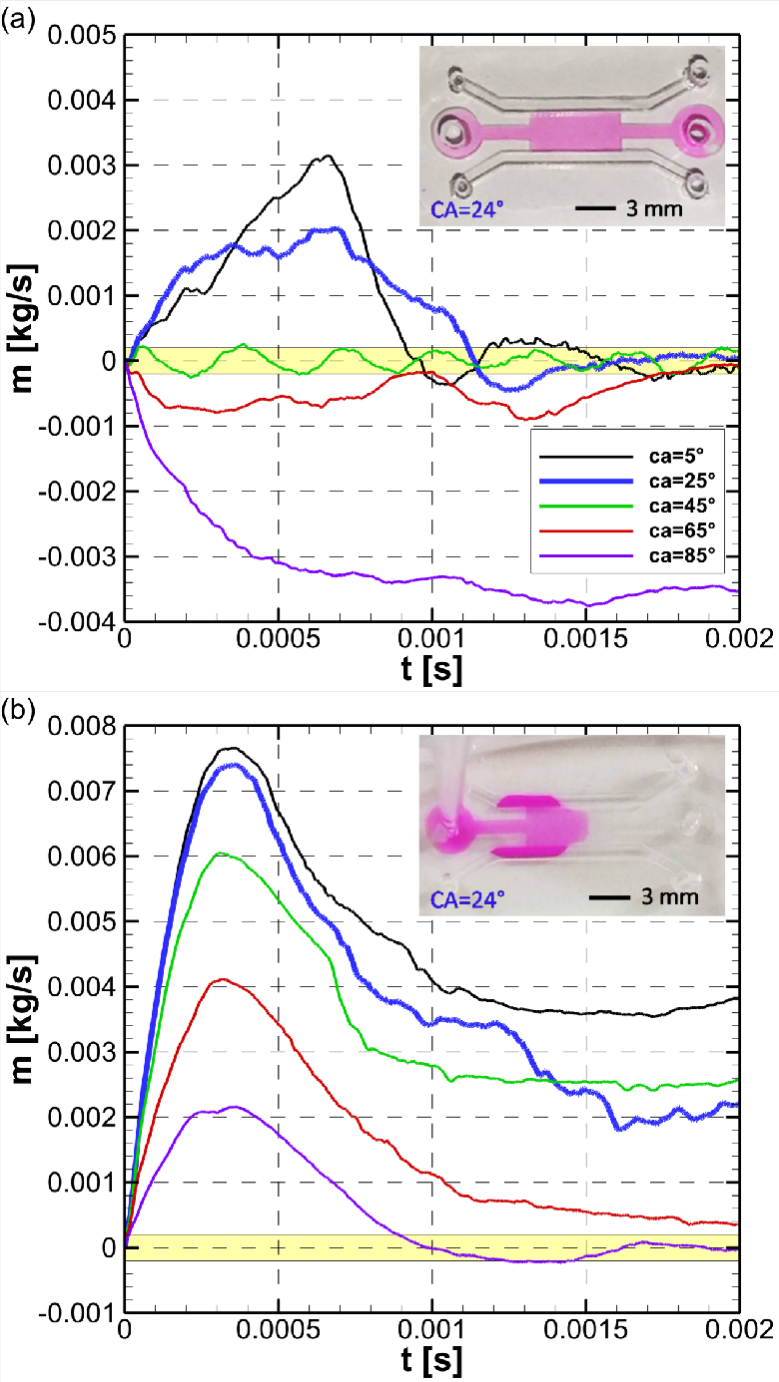
Effect of contact angle of the bottom wall on the inlet mass flow rate at (a) 0 and (b) 1000 Pa inlet pressure. *h* = 50 μm, *H* = 200 μm, *r* = 5 μm, and *P*_in_ = 0 Pa. (insets) Experimental results of water confinement tests within the STICI3Ds: (a) confined at *P* = 0 Pa inlet pressure and (b) nonconfined under high inlet pressures. See the corresponding Movies S2 and S7 in the Supporting Information.

On the one hand, if the contact angle reduces on the bottom wall, the surface becomes more hydrophilic, and the force that pulls the interface to side channels increases, which may cause the liquid to leak or overflow to the side channels. On the other hand, if the contact angle of the bottom wall increases, the force pulling the liquid decreases, and the fluid is not drawn into the trench. The effect of the contact angle of the bottom wall on the inlet mass flow rate and the condition of liquid confinement is shown in Figure 8.

The results in Figure 8a show that, as the contact angle increases, the mass flow rate from the inlet reduces, and for contact angles greater than 45°, the water retreats from the trench where it initially resides back to the trench inlet. When the liquid is pushed from the inlet channel with 1000 Pa, the liquid overflows to the side channel for most contact angles as shown in Figure 8b. As the contact angle increases, the forces that pull the interface to side channels decrease, and for contact angles over 85°, the water is confined within the trench for inlet pressures under 1000 Pa. Considering the results shown in Figure 8, using PDLcG rather than plain glass yields a more robust confinement for liquid loading at 0 Pa inlet pressure, due to its lower contact angle.

The condition of confinement under 0 Pa and high-pressure loading cases was investigated experimentally as well. As described in a previous section (see the section Mold and Device Fabrication), in order to load liquid with higher pressure, STICI3Ds were fabricated with narrower inlet diameters. When the liquid was pushed gently and slowly, the liquid was confined within the shallow trench (see Movie S6 in the Supporting Information). However, when the liquid was pushed by the micropipette faster and stronger, the liquid overflowed to side channels (see Movie S7 in the Supporting Information). The experimental results of confined and overflown cases are also shown in Figure 8.

### Effect of Hydrophilic Surface Selection

At the current state of the study, a feasible chip design was developed after the selections of proper channel height and bottom wall material and after showing that the manufacturing tolerance at the channel corners does not affect the liquid confinement condition. However, in the mass production of the microfluidic devices, failures such as unplanned high inlet pressures or manufacturing defects might occur. In order to make the design more resilient against liquid overflow under such failures, the selection of hydrophilic surfaces was further investigated.

**Figure 9:**
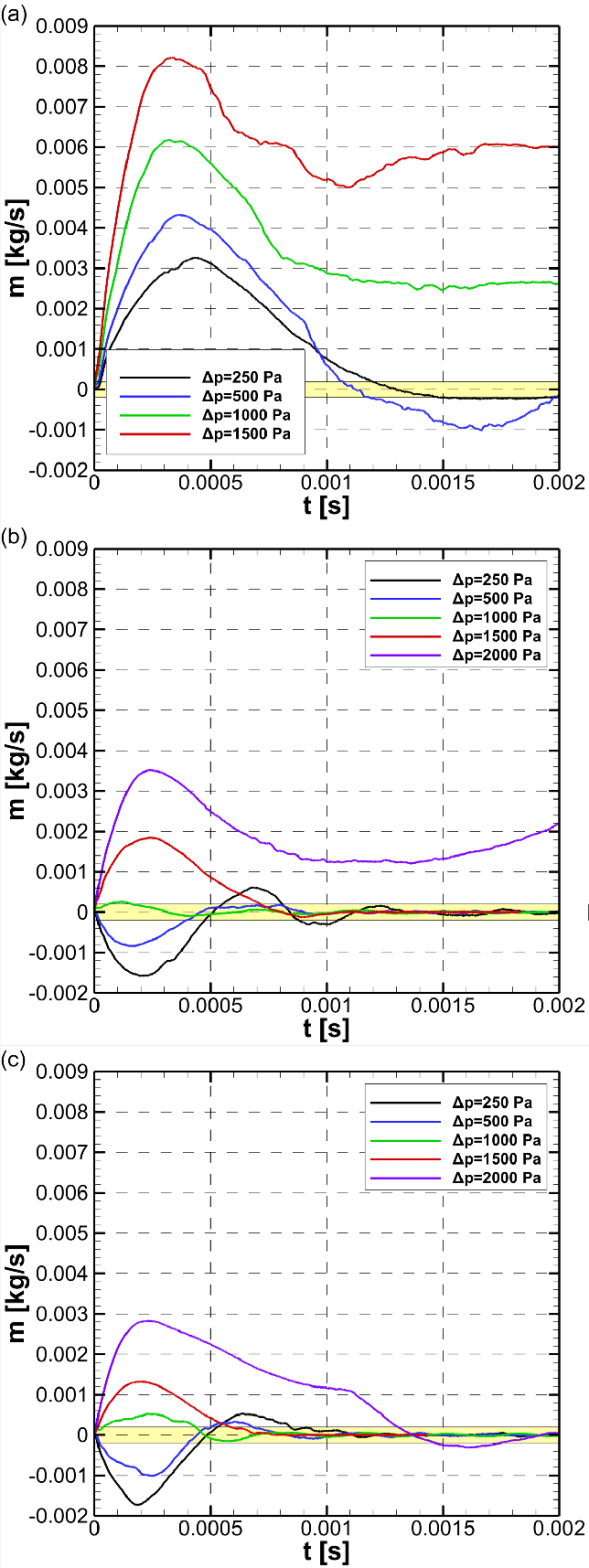
Effect of the selection of hydrophilic surface and corner radii on the inlet mass flow rate under various inlet pressures. *h* = 50 μm, *H* = 200 μm, (a) plain glass whole bottom wall, *r* = 5 μm, (b) plain glass midbottom wall, PDMS side-bottom wall, *r* = 5 μm. (c) Plain glass midbottom wall, PDMS side-bottom wall where corners left sharp (*r* = 0 μm).

Instead of using hydrophilic surfaces for all bottom walls, the bottom wall of the side channel was selected as PDMS, while the bottom wall of the trench was selected as plain glass. The zones on the bottom wall where plain glass and PDMS materials were assigned are shown in Figure 1c in red and blue, respectively. In both cases, where plain glass was assigned for the whole bottom wall and where plain glass and PDMS were assigned for the bottom wall of the trench and side channels, they were numerically tested under various inlet pressures, since both designs successfully confine the water within the trench under 0 Pa inlet pressure. The inlet mass flow rates of these designs under various loading pressures are shown in Figure 9.

It can be seen from Figure 9a that water could not be confined for the design with plain glass at the whole bottom wall with inlet pressure over 500 Pa. However, water was confined up to 1500 Pa inlet pressure for the case with PDMS bottom wall for the side channels as shown in Figure 9b. These results clearly show that coating the bottom wall of the side channels with a hydrophobic material such as PDMS substantially reduces the risk of liquid overflow into the side channels. The effects of the corner radius on water confinement were shown in the previous subsection; however, it was not tested under an inlet pressure higher than 0 Pa. The effects of the sharp corner on the inlet mass flow rate for various inlet pressures are shown in Figure 9c. The comparison of Figure 9b with 9c shows that a design with sharp corners successfully confines the water under 2000 Pa inlet pressure, where the design with rounded corner fails over 1500 Pa. However, as stated before the effect was minor compared to other design parameters, and no extra measures were taken to make the corners of the trench sharper.

After a numerical investigation on the effects of chosen parameters, the ideal design of the STICI3D was identified to have a trench height *h* of 50 μm and a side channel height *H* of 200 μm with PDLcG on the whole bottom wall, even though using hydrophilic surfaces only at the trench bottom wall reduces the risk of liquid overflow into the side channels, for the sake of simplicity in fabrication.

### Collagen Confinement Tests on Resulting STICI3D

A STICI3D with a trench height *h* of 50 μm and side channel height *H* of 200 μm was loaded with hydrogel solution dyed with Rhodamine B as explained in a previous section (see the section Preparation and Formation of Hydrogel), and the hydrogel was imaged by an inverted fluorescence microscope (Zeiss Axio Observer Z1). The image over the whole area of the STICI3D was obtained by merging the tiles obtained from different locations over the STICI3D.

Figure 5 shows the successful confinement of hydrogel solution within the trench of the STICI3D. The areas in yellow indicate the locations of hydrogel dyed with Rhodamine B where side channels were open to air. Figure 5c is a bright-field image, whereas Figure 5d is a fluorescent-only image of the STICI3D. Figure 5e displays a merged image of the overlapping bright-field and fluorescent image.

In order to show the 3D profile of the hydrogel, the samples were also imaged by a Leica DMI8 SP8 confocal microscope with the z-stack function, and the 3D view was reconstructed by LAS X software (Figure 2b). The height of the hydrogel confined within the trench was measured as 52 μm, which is almost the same as the desired trench height (*h* = 50 μm).

### Diffusion Test through the Hydrogel in STICI3D

**Figure 10:**
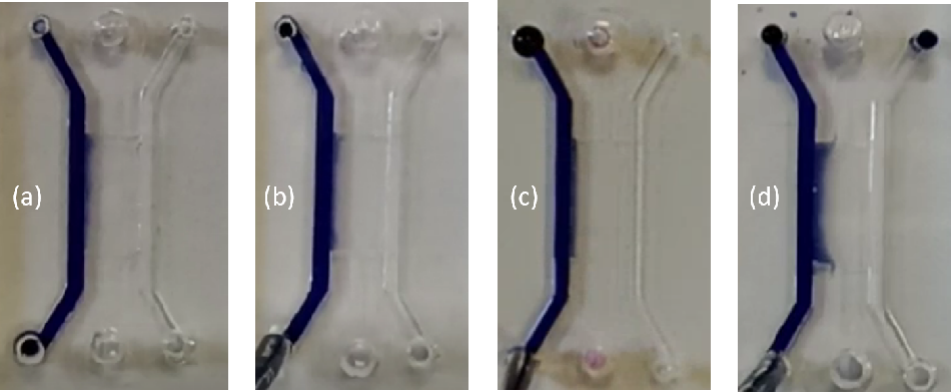
Diffusion of dyed water through the hydrogel inside the shallow trench under constant flow rates. Mean velocity of the dyed water is (a) 0, (b) 1*V*, (c) 10*V*, and (d) 50*V*, where *V* = 185 μm/s.

To demonstrate the diffusion process through the hydrogel within the shallow trench of the STICI3D and the effect of flow rates on side channels on the diffusion process, four STICI3D chips were produced. For these chips, a collagen hydrogel solution was loaded, gelated under the shallow trench, and one of the side channels in each STICI3D was filled with water. In the first chip, dyed water was loaded to the empty side channel using a pipet. Dyed water at three different constant flow rates were infused using a syringe pump for the empty side channels of the three remaining chips. The image for each of the four chips was captured exactly 2 min after the liquid within the side channel reached the center of the side channel, and it is shown in Figure 10.

It can be seen that the dyed water diffuses through the hydrogel and that the side channels are capable of supporting laminar flow through them. Moreover the chemical concentration gradient through the hydrogel can be controlled via controlling the flow rate within the side channels.

### Cell-Based Assays Inside the STICI3D

#### Human Mesenchymal Cell Culture in Collagen

**Figure 11:**
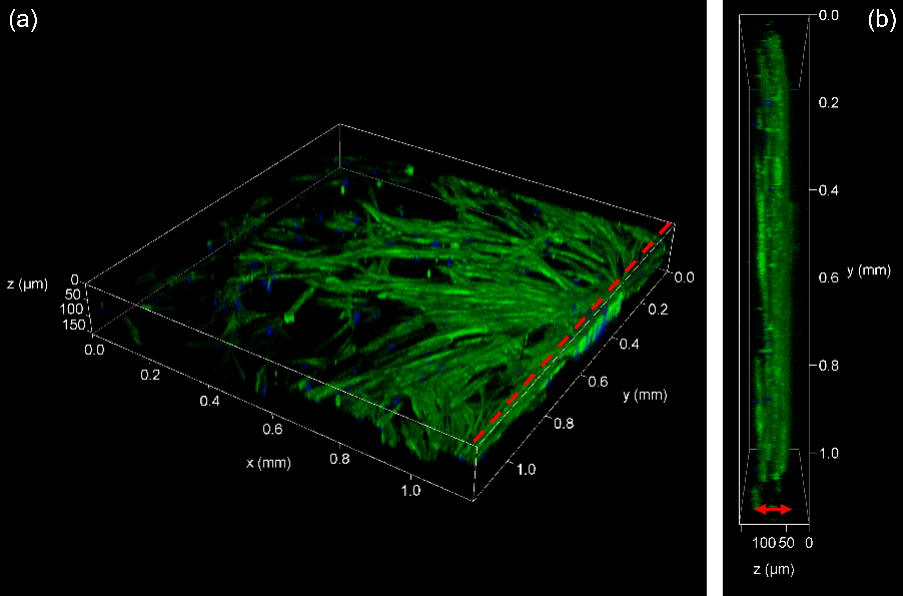
(a) 3D reconstruction of confocal microscope stack images of hMSCs grown in hydrogel within STICI3Ds (*h* = 50 μm, *H* = 200 μm), stained with phalloidin-FITC (green for actin proteins in the cytoplasm) and DAPI antifade solution (blue for cell nucleus). Red dashed lines indicate the hydrogel-side channel boundary where the left side is the hydrogel. (b) Side view of hMSC cells grown in hydrogel within STICI3Ds. Red two-sided arrow shows the hydrogel region.

Collagen hydrogel was prepared and confined within a STICI3D with a trench height *h* of 50 μm, and cells were seeded through one of the side channels (*H* = 200 μm). Immediately after the seeding, as described in the Cell Studies section, the cells were situated on the surface of the hydrogel facing the side channel onto which they attach and begin to proliferate. hMSCs interacted with the collagen and migrated into the hydrogel over time. Figure 11a shows a 3D reconstruction of confocal microscope z-stack images of hMSCs grown within the hydrogel in the STICI3D. The red dashed line indicates the hydrogel to side channel boundary where the left side of the dashed line is the trench filled with hydrogel. In Figure 11b, a side view of 3D-reconstructed confocal microscope stack images of hMSCs is presented. The two-sided red arrow indicates the total hydrogel region, which is ∼50 μm.

Similar reports of MSC proliferation are present for other microfluidic platforms.^41,43^ These results demonstrate that the proposed STICI3D platform can support and maintain the 3D culture of primary cells, such as hMSCs, for a period of 6 d. The data presented support our aim to produce a microfluidic platform that allows researchers to image cell growth and migration over extended periods in a biocompatible hydrogel.

#### Cellular Response of HUVEC on Collagen Hydrogel

**Figure 12:**
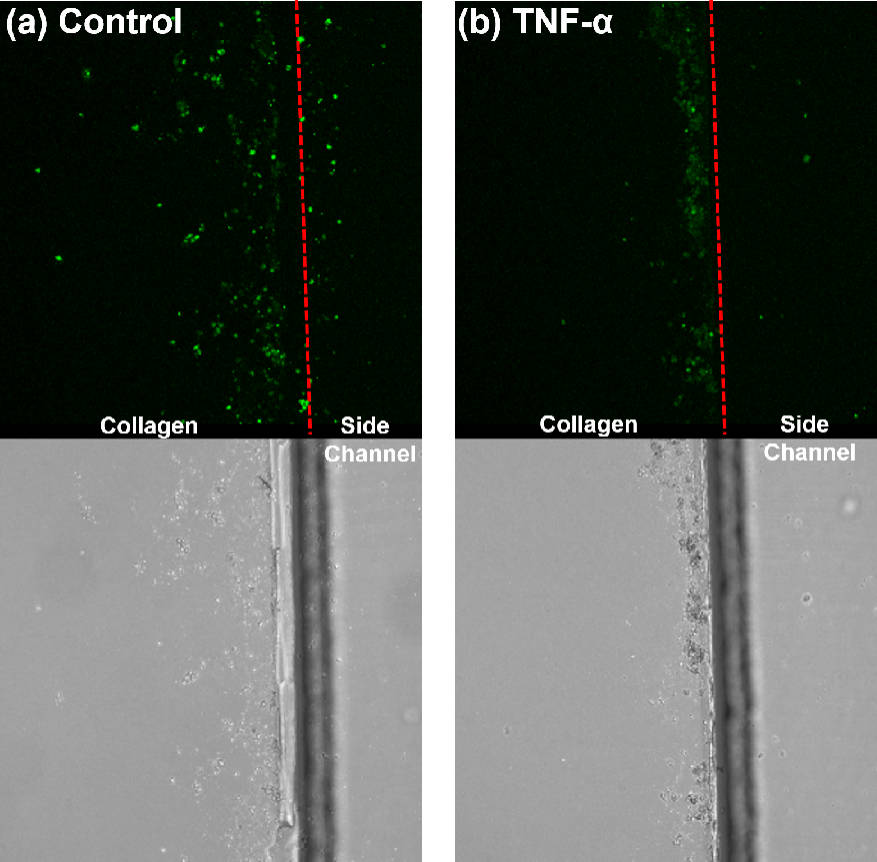
Experiment showing the functionality of STICI3D. The collagen was loaded onto the central channel as previously described, followed by the seeding of HUVEC cells (20 000 cells per middle channel). TNF-α was introduced at the channel opposite to the channel containing endothelial cells at 1 μg/mL concentration in the culture medium. After 24 h of incubation in the incubator, the live cells were imaged after cells were loaded with the live-cell imaging dye; calcein-AM. A Leica confocal microscope took images of live cells in the live-cell imaging mode under 5% CO_2_ and at 37 °C. (a) Control chip. (b) Chip with TNF-α added. This is representative of five different experiments, each with two chips. Dashed red line indicates the collagen-side channel boundary.

The functionality of the microfluidic STICI3D was assessed using endothelial cells placed on a collagen hydrogel, and the effect of adding TNF-α to the opposing side channel was investigated. TNF-α has been shown to exert an apoptotic effect on endothelial cells.^44^ In our STICI3D, TNF-α is added to the channel opposite to the seeded HUVECs. The 17.5 kDa protein readily passes through the hydrogel and affects HUVEC cells, causing cell death. We followed this by incubating the side channel of the STICI3Ds with TNF-α for 24 h and assessing cell viability by loading live HUVEC cells with calcein. In Figure 12, we show that 24 h of TNF-α incubation readily diminish viable HUVEC on the side channel, supporting the functionality of our STICI3D. These results in STICI3Ds indicate that our design permits a 3D cell culture of various cells; the three parallel channel conformation enables the delivery of various molecules across the hydrogel, the experiments being amenable to live-cell imaging.

## Conclusions

In vitro cell studies in microfluidic platforms are proven to be an effective method due to their ability to mimic, control, and monitor complex 3D in vivo microenvironments and low volume requirements for valuable cells and chemical solutions. The geometry and materials used for the fabrication of such microfluidic platforms play a vital role to yield a desired microenvironment, since the complex interaction of mechanical and biological phenomena that take place in microfluidic platforms is highly dependent on these factors. These microfluidic platforms are desired to be simple to fabricate, suitable for mass production, practical to use, and robust, which are achievable through a meticulous investigation of design parameters.

In our study, we designed a novel easy-to-fabricate microfluidic STICI3D platform with a simple design that allows the formation of 3D cell cultures inside and on the surfaces of a hydrogel matrix and precise control of the microenvironment through two side channels fully in contact with the side surfaces of the hydrogel confined within the shallow trench where high-resolution microscopic imaging of cells can be performed. The microenvironment within the hydrogel can be controlled by two adjacent side channels via diffusion and low-magnitude convection. In our design, the hydrogel solution can be loaded and confined within the trench through capillary action without necessitating micropillars. This shallow trench-induced design allowed us to develop a simple, easy-to-fabricate device with a continuous and maximized surface area where chemicals within the side channels interact with the hydrogel. Our simple design is not only easy to fabricate through soft lithography techniques using a mold made with adhesive tapes and razor but also more suitable for mass production methods. In order to identify the effect of each design parameter, we numerically investigated the effects of several geometric parameters (channel height, corner radii) and surface properties (the locations and wettability of the surfaces) on the confinement of hydrogel solution within the trench of the STICI3D using CFD simulations.

We simplified the geometry of a numerical model to a 2D cross-section of the STICI3D thanks to its simple geometric form and simulated the behavior of an initially confined liquid within the trench under various inlet pressures for different configurations. We employed a numerical setup compatible with similar flow problems reported in our previous studies, and we validated our numerical results by comparing them with experiments. We have shown that the STICI3D design with a trench height *h* of 50 μm, side channel height *H* of 200 μm, and PDLcG at the bottom wall is the optimum configuration for the successful confinement of hydrogel solution within the trench and for fabrication simplicity. With confocal microscopy results we demonstrated that hMSCs can readily grow and proliferate in 3D within collagen hydrogels formed within the shallow trench, supporting the functionality of our novel STICI3D platform. We also demonstrated that HUVECs lining the collagen respond to the molecules diffusing through the hydrogel.

The easy-to-fabricate nature and simple design of the STICI3D platform makes it suitable for researchers interested in fabricating custom PDMS devices as well as those who are in need of ready-to-use plastic platforms. As such, the STICI3D in vitro platform has great potential in imaging cell–cell interactions of multiple cells, angiogenesis, vasculogenesis, and semiquantitative analysis of drug response in cells.

## Supporting Information Available

Water confinement test movie of STICI3D with *h* = 50 μm: before hydrophobic recovery (MP4)
Water confinement test movie of STICI3D with *h* = 50 μm: confinement (MP4)
Water confinement test movie of STICI3D with *h* = 100 μm: confinement (MP4)
Water confinement test movie of STICI3D with *h* = 150 μm: confinement (MP4)
Water confinement test movie of STICI3D with *h* = 300 μm (MP4)
Water confinement test movie of STICI3D with *h* = 50 μm: pressured inlet–confined case (MP4)
Water confinement test movie of STICI3D with *h* = 50 μm: pressured inlet–overflown case (MP4)
Mold production demo of STICI3D (MP4)
Experimental validation details (Design feature dimensions of the STICI3D, mold production, profilometer measurements, contact angle measurements, water confinement tests, cell isolation) and Numerical results (Effect of corner radius and trench height) (PDF)
