# Supplementary material for: An Easy-to-Fabricate Microfluidic Shallow Trench Induced Three-Dimensional Cell Culturing and Imaging (STICI3D) Platform
Source: ACS Omega. 2022 Mar 2;7(10):8281–93. doi: 10.1021/acsomega.1c05118 (PMC8928507; doi:10.1021/acsomega.1c05118)
Supplement: Supplementary file 9 — ao1c05118_si_009.pdf [file ao1c05118_si_009.pdf]

Supplementary Information

An easy-to-fabricate microfluidic shallow trench induced three-dimensional cell culturing and imaging (STICI3D) platform

Umut Can Coskun,[†] Funda Kus,[‡] Ateeq Ur Rehman,[¶] Berna Morova,[§] Merve Gulle,[‖] Hatice Baser,[‡] Demet Kul,[⊥] Alper Kiraz,[§,#] Kemal Baysal,[⊥,@] and Ahmet Erten [*‖]

[†] Faculty of Aeronautics and Astronautics, Istanbul Technical University, Turkey.
[‡] Department of Biomedical Sciences and Engineering, Koç University, Turkey.
[¶] Biomedical Engineering Technology Program, Foundation University Islamabad, Pakistan.
[§] Department of Physics, Koç University, Turkey.
[‖] Department of Electronics and Communication Engineering, Istanbul Technical University, Turkey. [*] *E-mail: aerten@itu.edu.tr*
[#] Department of Electrical and Electronics Engineering, Koç University, Turkey.
[⊥] School of Medicine, Department of Biochemistry, Koç University, Turkey.
[@] KUTTAM Research Center for Translational Medicine, Koç University, Turkey.

Contents

1 Experimental Validation **S2**
1.1 Design feature dimensions of the STICI3D . . . S2
1.2 Mold production . . . S2
1.3 Profilometer measurements . . . S4
1.4 Contact angle measurements . . . S5
1.5 Water confinement tests . . . S6
1.6 Cell isolation . . . S6
1.7 Supplementary Movies . . . S7

2 Numerical Results **S8**
2.1 Effect of corner radius . . . S8
2.2 Effect of trench height . . . S8

1 Experimental Validation

1.1 Design feature dimensions of the STICI3D

Figure S1 shows the 2D sketch of the design features of the STICI3D microfluidic platform used in this study. The dashed blue circle on the figure indicates the puncher perimeter for the inlet chamber.

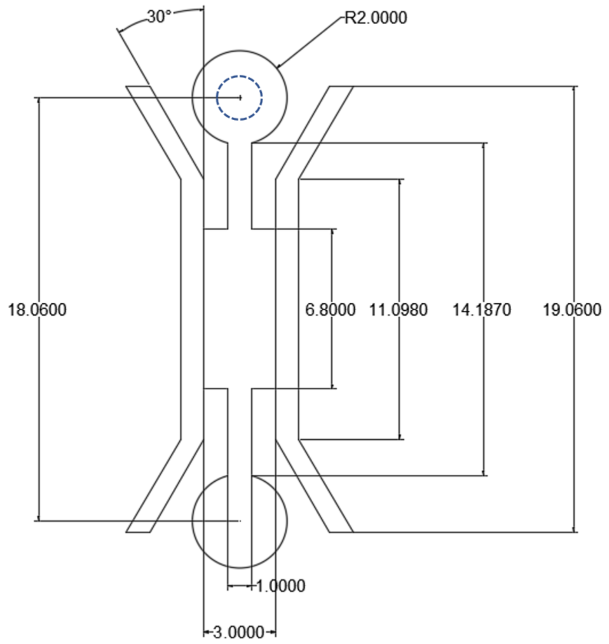

Figure S1 2D annotated sketch of the STICI3D design. The dashed blue circle indicates the puncher perimeter.

1.2 Mold production

The STICI3D platform was designed to have a trench in its middle fully in contact with two side channels, such that the trench has a width of 3 mm and each of the side channels are 1 mm in width. The length of the trench was designed to be 6.8 mm. Shallow trench and resulting microchannels over the PDMS part were produced by a combination of two types of adhesive tapes: Kapton and Aluminum, having an inherent thickness of 50 μm and 150 μm, respectively. Kapton tape was used for obtaining the trench height of **h**=50 μm, whereas a total height of **H**=200 μm was obtained by placing the Aluminum tape over the Kapton tape for the side channels.

As sketched in the Figure S2, both layers were attached over a piece of acetate paper to provide a smooth surface for the PDMS part that will be bonded to PDLcG. The acetate paper was placed over a plastic Petri dish with the help of a double-sided tape to keep it stable in its place. Laid tapes were cut either manually by a razor or by a digital craft cutter device (Silhouette Cameo, Silhouette America Inc.) separately for each layer of Kapton and Aluminum tape. Standard Ratchet blade with blade number “1” was used for

the Kapton tape, and blade number "2" was used for the Aluminum tape with thickness parameter selected as "31" for both, where cut-types were selected as "single-cut" for the Kapton tape and "double-cut" for the Aluminum tape. Movie S8 displays a demo of the mold production. Figure S3 shows a magnified image of the fine cut obtained for both tape types. In addition, Figure S4 shows a photograph of the produced mold.

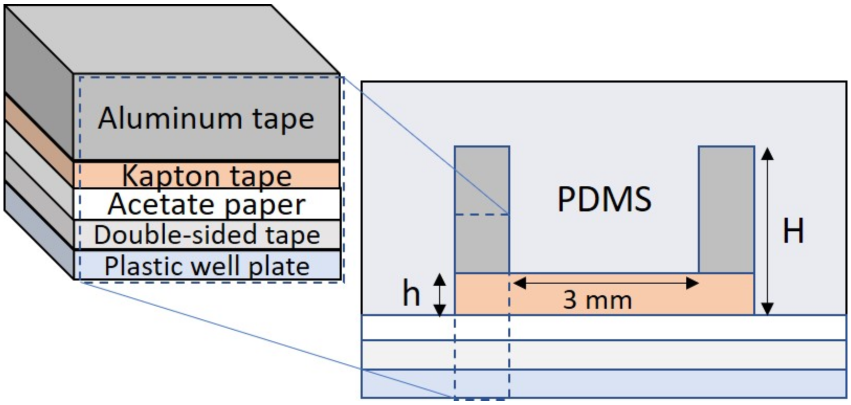

Figure S2 Cross-sectional view of the mold for the STICI3D with h=50 μm. Colored parts indicate the material used for each layer.

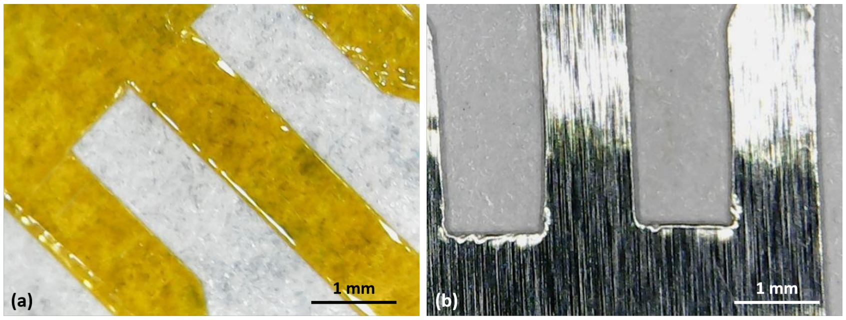

Figure S3 Examples of cuts by Silhouette Ratchet blade: (a) Kapton (b) Aluminum tape.

Same cutting procedure was applied for designs with increasing trench height by only changing the number of layers and/or the tape order for the purpose of obtaining the desired height. Table S1 shows the tapes used for the top and bottom layers for producing various heights. Figure S4 (a) and (b) are top-view pictures of the molds with **h**=50 μm and **h**=100 μm trench heights, respectively.

Table S1 Tapes used in producing molds with various trench heights.

**Trench height (h)**	**Material**
50 μm	single-layer Kapton tape
100 μm	double-layer Kapton tape
150 μm	single-layer Aluminum tape
300 μm	double-layer Aluminum tape

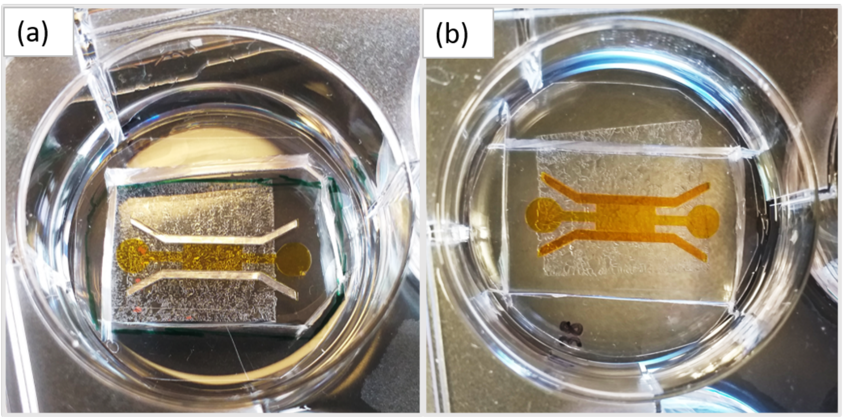

Figure S4 Photographs of the molds produced with (a) single- and (b) double-layer Kapton tape to form the trench.

The real time mold fabrication process which takes only around 4 minutes can be seen in Movie S8 . To demonstrate the reusability of the molds, a single mold was used five times to cast PDMS and confinement tests were performed for STICI3D devices obtained from PDMS parts demolded from this same mold. Figure S5 shows the mold after repeated usage and inserted figures show the water confinement within the trench.

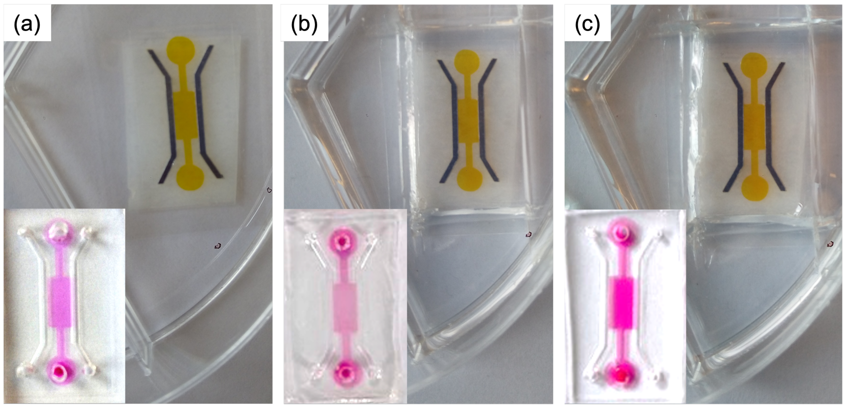

Figure S5 Mold reusability test (a) Mold after 1st casting (b) after 3rd casting (c) after 5th casting for STICI3D devices with h=50 μm. Inserted subfigures show the dyed water confinement results within the trench.

1.3 Profilometer measurements

In order to validate the channel heights, channel profiles of the STICI3D were measured with a Dektak profilometer over both the PDMS part and the mold. The surfaces of the demolded PDMS part were scanned with a 12.5 μm stylus radius and 1 mg Stylus force for a length of 6500 μm and duration of 90 s over two different locations. Similarly, the surface of the mold was scanned. Profilometer results (Figure S6) prove that the intended trench and side channel height can successfully be obtained with an approximate trench (**h**=50 μm) and side channel height (**H**=200 μm). Standard deviation for the data shown in part **A** of the PDMS chip location-1 and location-2 are 0.19 μm and 0.81 μm, respectively. For parts **B**, same analysis gives standard deviation values for location-1 and location-2, as 0.26 μm and 0.07 μm, respectively. Table S2 compares the intended (design) and measured (profilometer) dimensions of the STICI3D device.

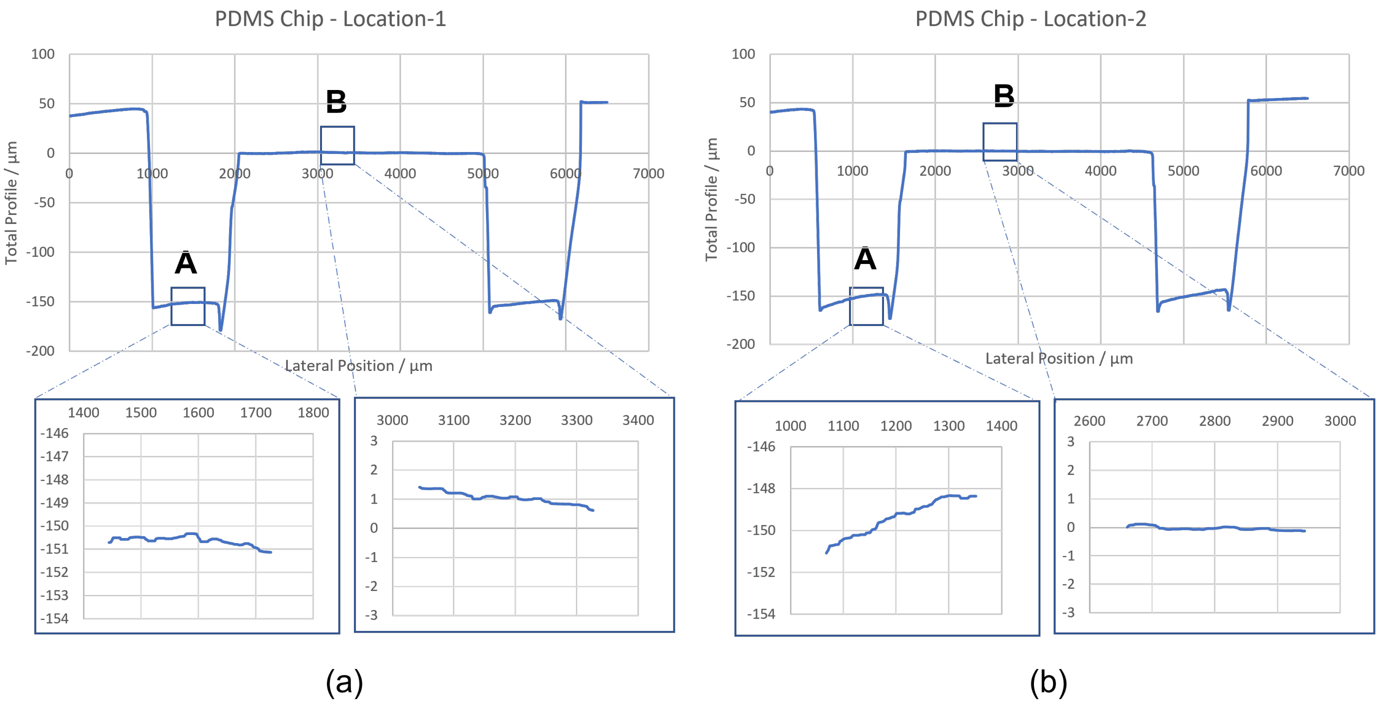

Figure S6 Dektak profilometer measurement of the PDMS part of STICI3D with h=50 μm trench height recorded over two different locations. Inserted images on bottom figures show magnified view of parts A and B.

Table S2 Designed and measured dimensions of the produced STICI3D devices.

	Designed	Measured
**h (μm)**	50	48
**w (μm)**	3000	2910
**H (μm)**	200	195

1.4 Contact angle measurements

All contact angles at the liquid–solid interface were measured by static sessile drop method under ambient laboratory conditions. Image of the liquid drop was captured by a camera mounted to the optical system with a backlight. The contact angle of deionized water and collagen solution was investigated by separately placing a drop of 3 μl volume over the surface of plain glass (Microscope slides, ISOLAB Laborgeräte GmbH), PDL-coated-glass (PDLcG; Menzel Gläser Polysine slides, Thermo Scientific), PDMS-coated-glass and PDMS demolded from Kapton tape in a medium of air. Images were captured with a camera and S-Eye software (Figure S7). Then, they were analyzed with the contact angle tool of ImageJ software. As listed in Table S3, the contact angles of DIW and collagen solution were found to be quite close to each other for all three surfaces . The contact angle of collagen solution on PDMS was found to be the highest of all three, indicating a hydrophobic behavior, whereas PDLcG showed lower contact angle for both water and collagen solution in comparison to plain glass. Furthermore, the water contact angle on the PDMS surface was measured after exposing the surface to Oxygen plasma (60 s under vacuum, with 100 W RF power) which renders the surface substantially more hydrophilic. The water contact angle of PDMS was recorded as 18° immediately after plasma, which is a drastic decrease from the contact angle measured over PDMS surface before plasma exposure. Similarly, the water contact angle of PDLcG was recorded as less than 5° after surface treatment with Oxygen plasma.

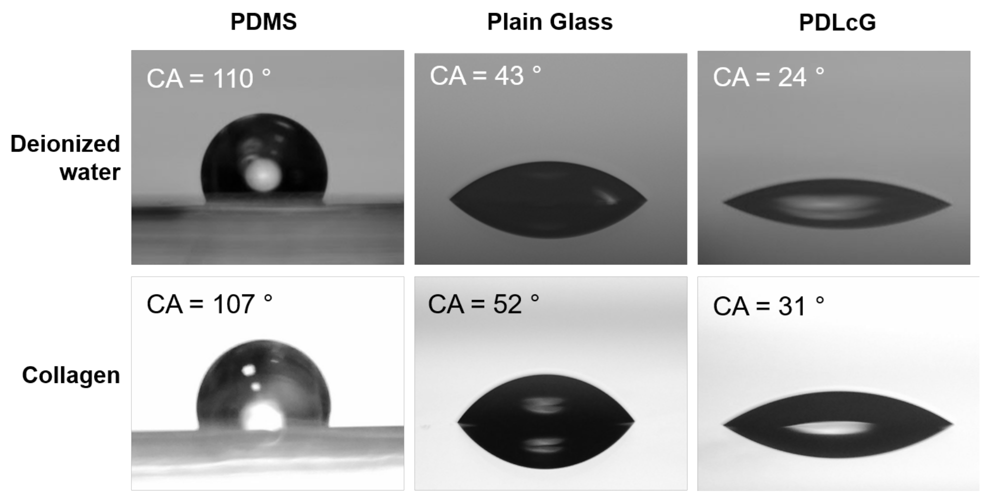

Figure S7 Sessile drop contact angle images of DIW and collagen over plain glass, PDLcG, and PDMS.

Table S3 Contact angles of DIW and collagen over plain glass, PDLcG, and PDMS.

Surface material	Deionized water contact angle	Collagen contact angle
PDMS	110 °	107 °
PDMS demolded over Kapton	111	-
Plain glass	43 °	52 °
PDLcG	24 °	31 °

1.5 Water confinement tests

The effect of wettability of the walls within a STICI3D was also investigated experimentally with a trench height **h**=50 μm. In this experiment, confinement of water within the trench was tested for STICI3Ds both before and after hydrophobic recovery (See Movies S1 and S2). It was observed that in both configurations the water was successfully confined within the trench. However, in the STICI3D tested before hydrophobic recovery, even though water was not overflowing to side channels, it was leaking and wetting the surfaces of the side channels (See Figure S8) due to lowered contact angles of PDMS walls and PDLcG at the bottom. Therefore, the hydrogel solution was decided to be loaded into the STICI3D after hydrophobic recovery instead of a hydrophilic STICI3D since the former does not require immediate use after assembly, rather it can be used anytime after hydrophobic recovery following the manufacturing.

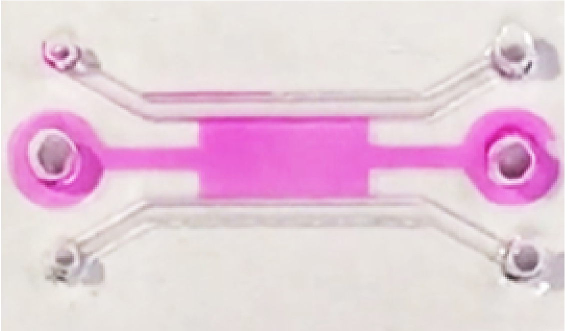

Figure S8 Water confinement inside the STICI3D with trench height h=50μm, immediately after plasma treatment. Areas filled with Rhodamine B solution in deionized water are seen as pink. Continuous recording of the STICI3D during loading is demonstrated in Movie S1.

1.6 Cell isolation

The hMSCs used in this study were isolated from umbilical cords obtained during childbirth. Dokuz Eylul University Non-Interventional Research Ethics Committee approved the use of human biological material with 3012-GOA protocol and decision number 2016 / 29-18. Informed written consent was obtained from all women whose cords were used in this study. In our study, pregnant women who underwent elective cesarean sections at Health Sciences University, Tepecik Training and Research Hospital in Izmir were included. The sterile cleaned cord was placed in 4 °C sterile phosphate buffer solution (PBS) (PAN-Biotech, Aidenbach, Germany) containing

100 U/mL penicillin and 100 μg/mL streptomycin for preservation and immediately transferred to Dokuz Eylul University, Faculty of Medicine, Medical Biochemistry Department, cell culture laboratory.

1.7 Supplementary Movies

Movie S1: Water confinement test movie of STICI3D with **h**=50 μm: before hydrophobic recovery - leakage
Movie S2: Water confinement test movie of STICI3D with **h**=50 μm: confinement
Movie S3: Water confinement test movie of STICI3D with **h**=100 μm: confinement
Movie S4: Water confinement test movie of STICI3D with **h**=150 μm: confinement
Movie S5: Water confinement test movie of STICI3D with **h**=300 μm
Movie S6: Water confinement test movie of STICI3D with **h**=50 μm: pressured inlet - confined case
Movie S7: Water confinement test movie of STICI3D with **h**=50 μm: pressured inlet - overflown case
Movie S8: Mold production demo of STICI3D

2 Numerical Results

2.1 Effect of corner radius

The effect of sharp corners and corners with a radius of **r**=5 μm on the inlet mass flow rate were compared in Figure S9.

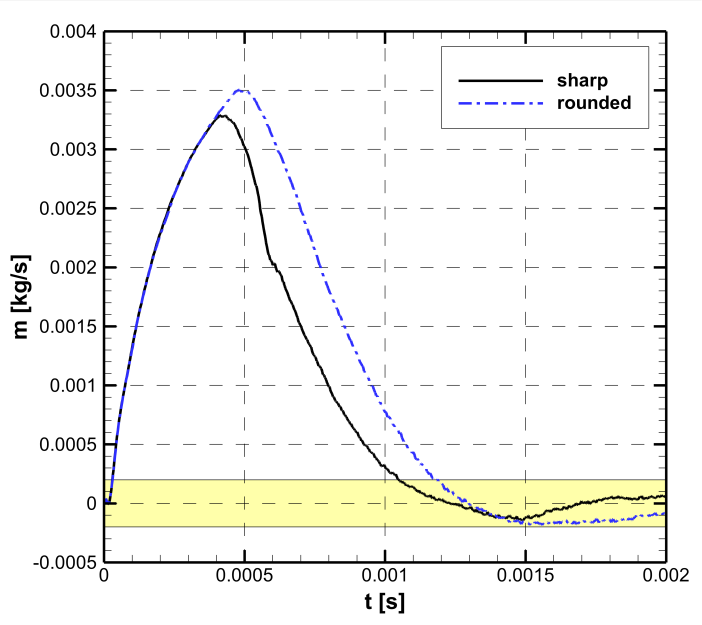

Figure S9 Effect of corner treatment on inlet water mass flow rate where h=50 μm, H=200 μm, P_{in}=0 Pa, PDLcG on middle and side bottom wall. The vicinity of zero mass flow rate indicating liquid confinement is highlighted in yellow.

2.2 Effect of trench height

The numerical results of the effect of trench height are shown in Figure S10. It can be seen that for **h**=25 μm and **h**=50 μm, the water is sucked into the channel and is confined. On the other hand, for **h**=200 μm and over, the liquid is pushed back to the inlet channel.

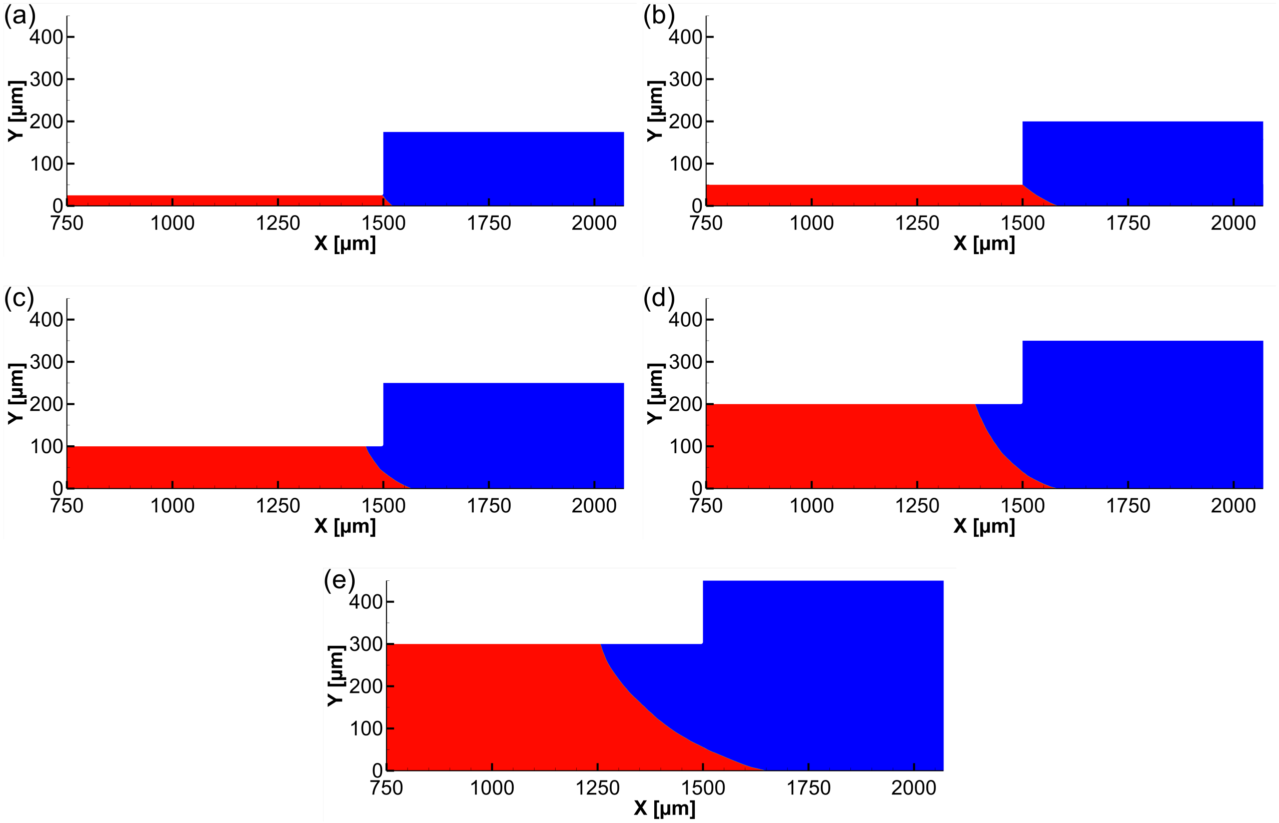

Figure S10 Resulting air-water interface shapes at t=0.002 s for (a) h=25 μm, (b) h=50 μm, (c) h=100 μm, (d) h=200 μm, (e) h=300 μm where H=h+150 μm, r=5 μm, P_{in}=0 Pa, PDLcG on trench and side bottom walls.
